# Genomic assessment of invasion dynamics of SARS-CoV-2 Omicron BA.1

**DOI:** 10.1126/science.adg6605

**Published:** 2023-07-20

**Authors:** Joseph L.-H. Tsui, John T. McCrone, Ben Lambert, Sumali Bajaj, Rhys P.D. Inward, Paolo Bosetti, Houriiyah Tegally, Verity Hill, Rosario Evans Pena, Alexander E. Zarebski, Thomas P. Peacock, Luyang Liu, Neo Wu, Megan Davis, Isaac I. Bogoch, Kamran Khan, Meaghan Kall, Nurin Iwani Binti Abdul Aziz, Rachel Colquhoun, Áine O’Toole, Ben Jackson, Abhishek Dasgupta, Eduan Wilkinson, Tulio de Oliveira, The COVID-19 Genomics UK (COG-UK) consortium, Thomas R. Connor, Nicholas J. Loman, Vittoria Colizza, Christophe Fraser, Erik Volz, Xiang Ji, Bernardo Gutierrez, Meera Chand, Simon Dellicour, Simon Cauchemez, Jayna Raghwani, Marc A. Suchard, Philippe Lemey, Andrew Rambaut, Oliver G. Pybus, Moritz U.G. Kraemer

**Affiliations:** 1. Department of Biology, University of Oxford, Oxford, UK.; 2. College of Engineering, Mathematics and Physical Sciences, University of Exeter, Exeter, UK; 3. Helix, San Mateo, USA.; 4. Institute of Ecology and Evolution, University of Edinburgh, Edinburgh, UK.; 5. Institut Pasteur, Université Paris Cité, CNRS, Paris, France.; 6. KwaZulu-Natal Research Innovation and Sequencing Platform (KRISP), Nelson R Mandela School of Medicine, University of KwaZulu-Natal, Durban, South Africa.; 7. Centre for Epidemic Response and Innovation (CERI), School for Data Science and Computational Thinking, Stellenbosch University, Stellenbosch, South Africa.; 8. Yale University, New Haven, USA.; 9. Department of Infectious Disease, Imperial College London, London, UK.; 10. UK Health Security Agency, London, UK.; 11. Google Research, Mountain View, USA.; 12. BlueDot, Toronto, Canada.; 13. Department of Medicine, Division of Infectious Diseases, University of Toronto, Toronto, Canada; 14. Pathogen Genomics Unit, Public Health Wales NHS Trust, Cardiff, UK.; 15. School of Biosciences, The Sir Martin Evans Building, Cardiff University, UK.; 16. Quadram Institute, Norwich, UK.; 17. Institute of Microbiology and Infection, University of Birmingham, Birmingham, UK.; 18. Sorbonne Université, INSERM, Institut Pierre Louis d’Épidémiologie et de Santé Publique (IPLESP), Paris, France.; 19. Big Data Institute, Li Ka Shing Centre for Health Information and Discovery, Nuffield Department of Medicine, University of Oxford, UK.; 20. Pandemic Sciences Institute, University of Oxford, UK.; 21. MRC Centre of Global Infectious Disease Analysis, Jameel Institute for Disease and Emergency Analytics, Imperial College London, London, UK; 22. Department of Mathematics, Tulane University, New Orleans, USA.; 23. Spatial Epidemiology Lab (SpELL), Université Libre de Bruxelles, Bruxelles, Belgium.; 24. Department of Microbiology, Immunology and Transplantation, Rega Institute, KU Leuven, Leuven, Belgium.; 25. Department of Pathobiology and Population Science, Royal Veterinary College, London, UK.; 26. Departments of Biostatistics, Biomathematics and Human Genetics, University of California, Los Angeles, Los Angeles, CA, USA.

## Abstract

SARS-CoV-2 variants of concern (VOCs) arise against the backdrop of increasingly heterogeneous human connectivity and population immunity. Through a large-scale phylodynamic analysis of 115,622 Omicron genomes, we identified >6,000 independent introductions of the antigenically distinct virus into England and reconstructed the dispersal history of resulting local transmission. We estimate that by the time Omicron BA.1 was reported in southern Africa (November 22^nd^, 2021) six of the eight largest transmission lineages were already established in England. During that time internationally well-connected hubs started acting as exporters of the variant which led to continued seeding of the VOC to England where it locally dispersed through the hierarchical travel network. Our results offer a detailed characterisation of processes that drive the invasion of an emerging VOC across multiple spatial scales. Genomic surveillance along the travelling network, coordinated and rapid decision making during the emergence of infectious diseases is necessary to delay their arrival.

## One sentence summary

Omicron variant was introduced into England before detection in South Africa and rapidly disseminated from city to city.

Since the emergence of SARS-CoV-2 in late 2019, multiple variants of concern (VOCs) have sequentially dominated the pandemic across the world. The Omicron variant (Pango lineage B.1.1.529 later divided into lineages including BA.1 and BA.2) was discovered in late November 2021, through genomic surveillance in Botswana and South Africa and a traveller from South Africa in Hong Kong (1) and designated a VOC by the World Health Organisation on 26 November 2021 (2). An initial surge in Omicron cases in South Africa indicated a higher transmission rate than previous variants (3), which studies later attributed to a shorter serial interval, increased immune evasion and greater intrinsic transmissibility (4–7). The mechanism for this greater transmissibility is hypothesised to be altered tropism and higher replication in the upper respiratory tract (8, 9). Together with waning levels of population immunity from previous infections and vaccination (10), local transmission of Omicron BA.1 was soon reported thereafter in major travel hubs worldwide, including New York City and London by early December 2021, despite travel restrictions on international flights from multiple southern African countries (11, 12).

Following the first confirmed case of Omicron BA.1 in England on 27 November 2021 (13), Omicron prevalence increased rapidly across all regions of England, with Greater London prevalence peaking first in mid-December at ~6%, followed by the South East region (14). Other metropolitan areas in the North West and North East saw similar but delayed increases in prevalence with observed peaks between early- and mid-January 2022. Incidence of Omicron BA.1 had declined substantially in Greater London and other southern regions by early-January 2022, resulting in a gradient of decreasing prevalence from north to south England (15). This spatiotemporal pattern of early spread was also observed for the Alpha variant in England (16), but is markedly different from that of the Delta variant, which spread initially from the North West and surrounding regions of England (17). Rapid growth in infections during the initial emergence of Omicron in England prompted the UK government to impose interventions including a move to “Plan B” non-pharmaceutical restrictions (a mandatory COVID pass for entry into certain indoor venues, face coverings and work-from-home guidance) on 8 December 2021 (18) and an accelerated program of booster vaccination for all adults by mid-December 2021 (19). The prevalence of SARS-CoV-2 in England decreased later in January 2022, coincident with a falling proportion of BA.1 infections as BA.2 lineage replaced BA.1 as the dominant lineage, which itself was later replaced by the BA.4 and BA.5 lineages (20–22).

Understanding and quantifying the relative contributions of the factors that determined the arrival and spatial dissemination of Omicron BA.1 in England can help inform the design of spatially-targeted interventions against future VOCs (23). Here, we analyse the initial Omicron BA.1 wave in England, using a dataset of 48,748 Omicron BA.1 genomes sampled in England. This dataset represents ~1% of all confirmed Omicron BA.1 cases in England during the study period and is combined with sub-city level aggregated and anonymized human mobility and epidemiological data from 313 lower tier local authorities (LTLAs) in England.

## International seeding events and Omicron BA.1 lineage dynamics

To investigate the timing of virus importations into England and the dynamics of their descendent local transmission lineages, we undertook a large-scale phylodynamic analysis of 115,622 SARS-CoV-2 Omicron genomes (BA.1/BA.2 and their descendent lineages), sampled globally between 8 November 2021 and 31 January 2022. About 42% (N=48,748) were sampled from England and sequenced by the COVID-19 Genomics UK (COG-UK) consortium (24). All available genomes (from COG-UK and GISAID (25) on 12 and 9 April 2022 respectively) collected before 28 November 2021 were included; genomes collected after that date were randomly subsampled in proportion to weekly Omicron case incidence while maintaining a roughly 1:1 ratio between English and non-English samples. To reduce potential bias caused by heterogeneous sequencing coverage, we performed a weighted subsampling of the English genomes using a previously developed procedure which accounts for variation in the number of sequences sampled per reported case at the Upper Tier Local Authority (UTLA) level (26) (supplementary material, materials and methods).

**Fig. 1:**
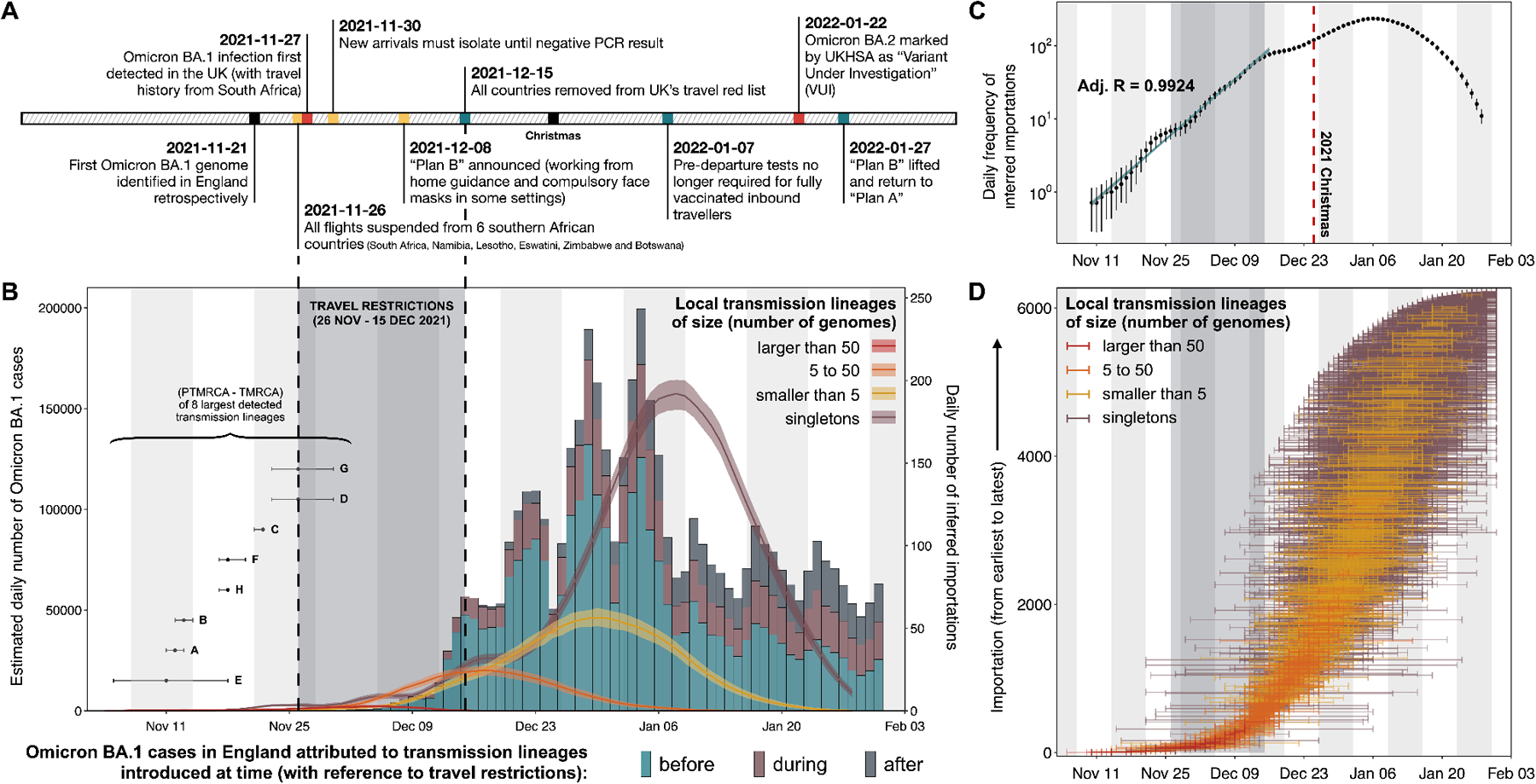
Dynamics of Omicron BA.1 transmission lineages in England. (A) Timeline of key events during the SARS-CoV-2 Omicron BA.1 wave in England until February 2022. (B) Histogram of the estimated daily number of Omicron BA.1 cases, coloured according to the proportion of cases attributable to importation at different times (shaded region shows period of travel restrictions). Solid lines represent the daily frequency of inferred importations (7-day rolling average), coloured according to the size of resulting local transmission lineages; shading denotes the 95% HPD across the posterior tree distribution. For each of the eight largest detected transmission lineages (labelled A to H), the estimated time of importation, TPMRCA (inferred time of parent of most recent common ancestor) and TMRCA (inferred time of most recent common ancestor) are shown in the bottom left of the panel. (C) Daily frequency of inferred importations (7-day rolling average), without stratification by size of resulting local transmission lineage (black dots); error bars denote the 95% HPD across the posterior tree distribution. Solid blue line represents the daily number of imports expected from an exponential model fitted to the observed 7-day rolling average importation intensity. (D) Distribution of TPMRCAs and TMRCAs of all 6,455 detected introductions. Each horizontal line represents a single introduction event that led to a transmission lineage or a singleton, with the left limit indicating the TPMRCA and the right limit indicating the TMRCA (or genome collection date for a singleton).

**Fig. 2:**
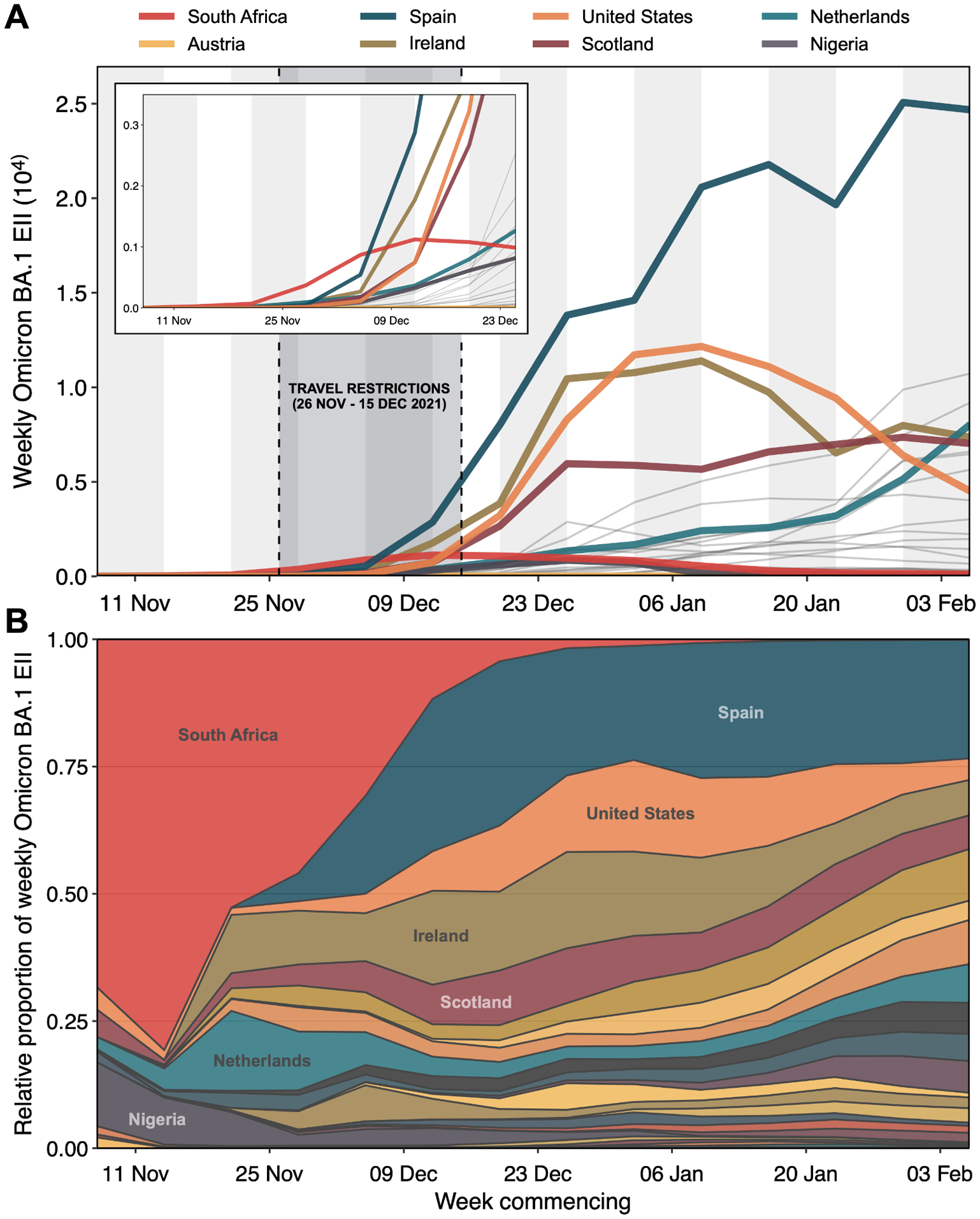
Estimated Importation Intensity (EII) of Omicron BA.1 from selected potential exporters. Estimated weekly number of Omicron BA.1 cases arriving in England from 27 countries (including Scotland and Northern Ireland independently) with the highest air passenger volumes arriving in England between November 2021 and January 2022 (collectively accounting for ~80% of total air passenger volume in this period). Thick solid lines represent EIIs from eight selected countries with notable contribution to the overall importation intensity at different points during the study period; thin grey lines represent all other countries. Inset shows a magnified view of early trends. (B) Relative proportion of weekly EII of Omicron BA.1 by country among selected potential exporters. Areas representing countries highlighted in (A) are labelled. See supplementary materials for sensitivity analyses.

We identified at least 6,455 [95% HPD: 6,184 to 6,722] independent importation events. Most imports from outside of England (69.9% [95% HPD: 69.0 to 70.7]) led to singletons (i.e., a single genome sampled in England associated with an importation event, which did not lead to observable local transmission in our dataset). The earliest importation event is estimated between 5 and 18 November (approximated as the midpoint between the inferred times of the most recent common ancestor (MRCA) of the transmission lineage and the parent of the MRCA (PMRCA)). Between the first introduction event and mid-December 2021, we infer an approximately exponential increase in the daily number of imports before a plateau in early January 2022 (Fig. 1C). There is some indication that the daily importation rate was raised between 22 November, when Omicron was first reported and the start of travel restrictions (Fig. 1C). Increased outflow of passengers before (and possibly in anticipation of) travel restrictions has been reported for earlier waves of SARS-CoV-2 (16, 27). This rapid growth in importation continued despite restrictions on incoming international travel from 11 southern African countries and could have originated from Omicron outbreaks in other countries in late November and early December 2021. To explore this hypothesis, we calculate the Estimated Importation Intensity (EII) of Omicron BA.1 from countries with the highest air traffic volumes to the UK capturing 80% of total air travel. We aim to increase the resolution of the global scale analyses in Tegally et al. (2023) (28) by focusing on Omicron imports to England specifically. For each source location, the EII measure combines the weekly average COVID-19 test positivity rate, weekly relative prevalence of Omicron BA.1 genomes, and monthly number of observed air passengers travelling to England (see supplementary material, materials and methods for details and sensitivity analysis using case data and geographic disaggregations; Fig S4–S6). While the earliest imports were mostly inferred to have come from South Africa, we observe a shift in Omicron BA.1 imports from South Africa to a larger set of countries, by late November/early December 2021 (Fig. 2), during the period of travel restrictions on South Africa. We performed a sensitivity analysis in which EIIs are instead calculated using per capita case incidence rather than test positivity and the results are broadly consistent (Fig. S6).

We conclude the exponential growth of BA.1 importations through mid-December is therefore in part due to introductions from countries other than South Africa (Fig. 1B and Fig. 2), which became major contributors due to the Omicron epidemics there and the substantial volume of air travel to England (Fig. S5). At the time when travel restrictions to 11 southern African countries were announced, sequences of Omicron BA.1 from only four countries had been uploaded to GISAID (25). We note that our work is not designed to quantitatively assess the impact of these restrictions on infection numbers in England.

To cross-validate the importation dynamics inferred from viral genomes using an independent data source, we extracted data from the Variant and Mutations (VAM) line list (29) provided by the UK Health Security Agency (UKHSA) and calculated the daily number of incoming travellers who were later tested positive for Omicron BA.1 in community surveillance (Pillar 2) of the UK SARS-CoV-2 testing programme. The temporal profile of importation intensity from these epidemiological data is broadly consistent with that inferred from the phylodynamic analysis, with the latter being temporally expanded and lagged compared to the former (Fig. S2). This observation is consistent with previous studies (30) and the apparent discrepancy represents the time lag between international importation and the first local transmission event that is observable from phylogenetic data.

As with the emergence of previous variants in England (17, 30), we find that transmission lineage sizes are overdispersed (Fig. S3), with most sampled genomes belonging to a small number of large transmission lineages. The eight largest transmission lineages (each with >700 genomes) together comprise >60% of the genomes sampled in England in our dataset (Fig. 1B). Most of these (six of eight) are inferred to have been imported before restrictions on travel from southern African countries were introduced (26 November 2021), and three could have been introduced before the first epidemiological signal of the new variant (an uptick in S-gene target failure, SGTF, samples identified by a private lab in South Africa on 15 November 2021 (Fig. 1B). Additionally, we observe a strong association between the size and time of importation of local transmission lineages, with most large transmission lineages attributed to early introductions between 5 and 13 November 2021 (Fig. 1B). This pattern can be recapitulated using a simple mathematical model; if all lineages share the same transmission characteristics, then the date of importation is the main determinant of transmission lineage size when the epidemic in the recipient location is growing exponentially (see materials and methods).

We estimate that 399 transmission lineages (including the eight largest) resulted from importation events before the end of restrictions on travel from southern Africa (15 December 2021); 29 of these lineages were introduced before 26 November 2021. Although these early imports account for only a small proportion (~6%) of the estimated total number of introductions, they are responsible collectively for ~80% of Omicron BA.1 infections reported in England to the end of January 2022.

Some transmission lineages from early importations were only detected several weeks after their inferred time of importation. However, we interpret this result cautiously, as we cannot exclude the possibility that these transmission clusters represent the aggregation of multiple independent transmission lineages, as a result of unsampled genetic diversity outside England. Such aggregation would result in earlier estimated dates of importation, potentially explaining the smaller than expected size (compared to predictions from simulations; Fig. S7 and Fig. S8) of these transmission lineages with unusually long importation lag (30). Future analyses incorporating detailed metadata on travel history could help reduce the degree of uncertainty in the number and timing of inferred importation events (31, 32).

## Human mobility drives spatial expansion and heterogeneity in Omicron BA.1 growth

The rapid increase in Omicron importations in late 2021 led to the establishment of local transmission chains, initially concentrated in Greater London and neighbouring LTLAs in the South West and East of England. This coincided with early increases in Omicron BA.1 prevalence in the corresponding regions, as observed from SGTF data and other epidemiological studies based on prevalence surveys (15). To further investigate the spatiotemporal dynamics of Omicron transmission lineages in England, we reconstructed the dispersal history of all identified transmission lineages (with >5 genomes) using spatially explicit phylogeographic techniques. Sampling of English genomes was highly representative of the estimated number of Omicron BA.1 cases at the UTLA level (Fig. S9; comparison with modelled case incidence with adjustment for changes in case reporting is shown in Fig. S10).

**Fig. 3:**
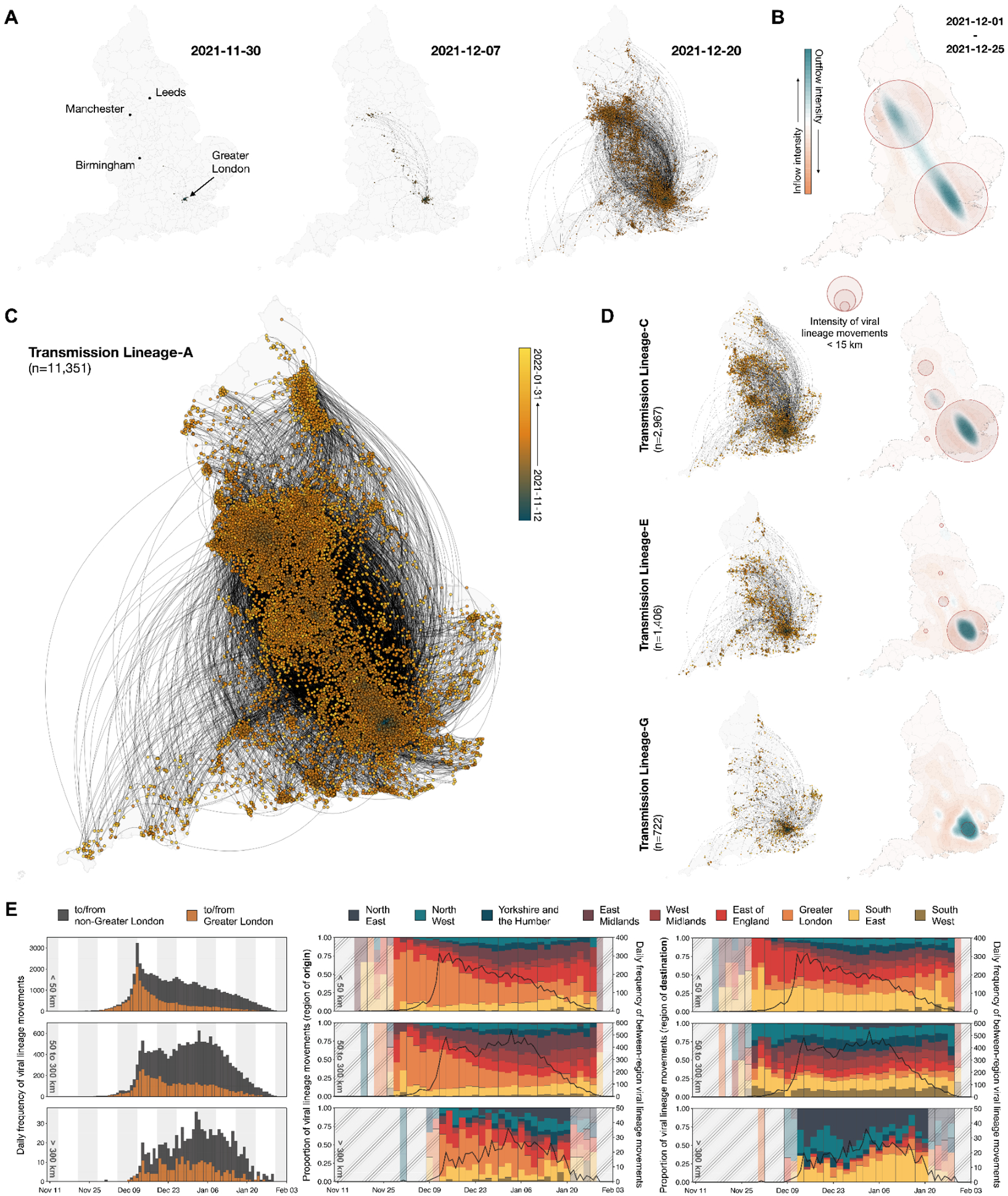
Spatiotemporal dynamics of Omicron BA.1 transmission lineages in England. (A and C) Continuous phylogeographic reconstruction of the dispersal history of the largest detected BA.1 transmission lineages in England (Transmission Lineage-A). Nodes are coloured according to inferred date of occurrence and the direction of viral lineage movement is indicated by edge curvature (anti-clockwise). Panel A shows the progress of dissemination at three specific times, and panel C shows the complete construction. (B) Geographical distribution of the intensity of inflow and outflow of viral lineages for Transmission Lineage-A from the beginning of December up to Christmas 2021. Blue colours indicate areas with high intensity of domestic lineage outflow; red colours indicate those with high intensity of inflow. Red circles indicate areas with high densities of local viral movements (distances <15 km); circle radii are proportional to that density. (D) Continuous phylogeographic reconstruction of Transmission Lineages C, E, and G (as per panel C) with corresponding maps of the geographical distribution of the intensity of viral lineage inflow and outflow (as per panel B). Fig. S12 provides an equivalent figure for Transmission Lineages B, D, F and H. (E) Plots in each row correspond to viral lineage movements across different spatial scales (top: <50 km, middle: 50 to 300 km, bottom: >300 km). (Left) Histograms show the daily frequency of viral lineage movements across spatial scales. Colours indicate whether the origin and/or destination of the viral lineage movements are inferred to have occurred in Greater London. (Middle/Right) Solid black lines represent the daily frequency of among-region viral lineage movements across spatial scales. Vertical bars indicate the proportions of viral lineage movements (aggregated at 2-day intervals);coloured according to their origin/destination locations. Shaded grey areas indicate periods when there were <9 inferred viral lineage movements per.

We observe multiple distinct stages to the spread of BA.1 across England, with the eight largest identified transmission lineages sharing broadly similar patterns of spatial dispersal. Unlike other variants, we find that the numbers of transmission lineages first detected are fairly evenly distributed among regions, with ~20% in Greater London (followed by 15.4% in the South East and 13.3% in the North West; if only introductions prior to December 2021 are considered, the value for Greater London is 27.3%). However, most of the early cases outside Greater London resulted in limited local spatial diffusion (Fig. 3, Fig. S11 and Fig. S12).

Further, initial long distance viral lineage movements from Greater London repeatedly arrived in multiple urban (according to 2011 Rural-Urban Classification by the UK Office of National Statistics (33)) conurbations in early/mid-December 2021, but local transmission was not established immediately. The fraction of all viral lineage movements that were local (within-city) remained between 25%−50% from December 2021 to January 2022 in all areas except Greater London and Greater Manchester. This fraction grew when local mobility levels recovered after the holiday period (34–37), coinciding with the time when local transmission was established across most LTLAs in England. In contrast, between November and December 2021, local viral movements in Greater London and Greater Manchester comprised ~90% and ~60% of all movements respectively, indicating that epidemics in those locations were driven by multiple locally-established lineages (Fig. S11). Further, cities other than Greater London acted primarily as sinks throughout the BA.1 wave with limited backflow of long-distance viral lineages from North West England to Greater London (e.g. Transmission Lineage-A and Transmission Lineage-B; similar dynamics are also seen for the South West of England; Fig. 3E). We define locations as sinks/sources according to whether there was a net flow of viral lineages into/out of the location over the study period.

Even after the establishment of local transmission in most English LTLAs, Greater London continued to be a source of mid-to-long range viral lineage movements (Fig. 3E). This is expected given that Greater London is a major travel hub in England’s mobility network (similar trends were observed during the Alpha wave in 2020 (16)). The importance of Greater London as a source of short range (<50 km) lineage movements declined through time (Fig. 3E, left-top) and we observe a secondary peak in the frequency of mid-to-long range movements (>50 km; Fig. 3E) driven predominantly by virus lineages emanating from the Midlands and southern England (Fig. 3E, middle and right). These observations are consistent with epidemiological data showing that most areas outside of southern England experienced a BA.1 incidence peak only in the last week of December 2021 or the first week of January 2022 (Fig. S13).

**Fig. 4:**
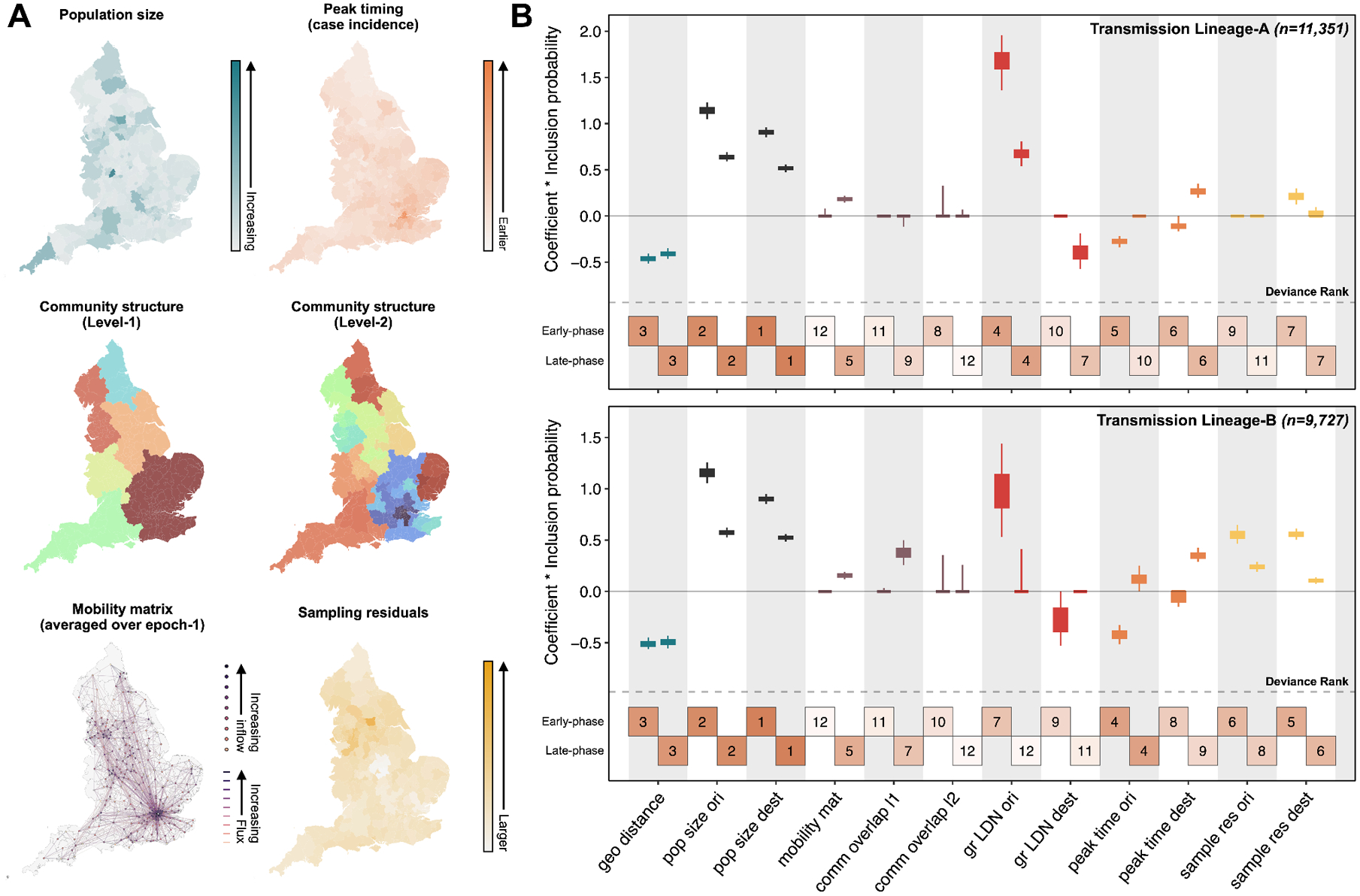
Predictors of Omicron BA.1 viral lineage movements in England. (A) Map at LTLA level of model predictors included in the discrete phylogeographic GLM analysis, for the largest detected BA.1 transmission lineages (Transmission Lineage-A). (B) For each predictor, the box and whiskers show the posterior distribution of the product of the log predictor coefficient and the predictor inclusion probability; the left hand value represents the expansion period estimate and the right hand value the post-expansion period estimate. Top panel shows estimates for Transmission Lineage-A and bottom panel shows those for Transmission Lineage-B. Posterior distributions are coloured according to predictor type: geographic distances (geo distance, dark blue), population sizes at origin and destination (pop size ori & pop size dest, black), aggregated mobility (mobility mat, purple), mobility-based community membership level 1 and level 2 (comm overlap l1 & l2, purple), Greater London origin and destination (gr LDN ori & gr LDN dest, red), time of peak incidence at origin and destination (peak time ori & peak time dest, orange) and the residual of a regression of sample size against case count regression at either origin and destination (sample res ori & sample res dest, yellow). Boxes at the bottom of each panel are numbered and shaded to represent the rank of predictors based on their deviance measure, with 1 indicating the largest (most important) and 12 indicating the smallest (least important).

To assess the contribution of demographic, epidemiological and mobility-related factors to the dissemination of Omicron BA.1 in England, we used a discrete phylogeographic generalised linear model (GLM) to test the association of those factors with viral lineage movements among LTLAs, across two periods (before 26 December 2021, and between 26 December 2021 and 31 January 2022; supplementary materials) (35, 36, 38). Using this time-inhomogeneous model we find evidence for a dynamic spatial transmission process, with change through time in the estimated effect sizes of most predictors (Fig. 4B). During the earlier “expansion” period of lineage transmission among cities, we observe consistently strong support for the gravity model components (a spatial interaction model in which travel intensity between pairs of locations increases with origin and destination population sizes but decreases with distance between them). Consistent with results from continuous phylogeography, the early period is characterised by directional viral dissemination; lineage movements tend to originate from Greater London (Fig 4b) and this is particularly pronounced for smaller transmission lineages (Fig. 3 and Fig. S12).

In three out of four analyses we also find greater dissemination out of LTLAs with earlier times of peak incidence during the expansion period and, conversely, a lower inflow of viral lineages during the post-expansion period in all analyses (Fig. 4 and Fig. S14). These results reflect the dynamic, network-driven nature of Omicron’s geographic spread, with variation in the timing of peak incidence reflecting heterogeneity in the underlying human mobility network, i.e. varying degrees of connection to locations with frequent early seeding events (39).

Interestingly, the human mobility matrix predictor is supported consistently only in the post-expansion phase (Fig. 4B), after local transmission had been established in most LTLAs. This reflects a transition from unidirectional long-distance movements to more homogeneous local viral lineage movements. Conversely, support for the gravity model predictors decreased over time (Fig. 4B), consistent with the notion that the gravity model better predicts city-to-city mobility and poorly describes diffusion-like mobility over short distances in urban areas (40). Importantly, the phylogeographic GLM results are consistent among the transmission lineages analysed (Fig 4B), and also when a simpler time-homogenous model is used (Fig. S15). These findings are consistent with our continuous phylogeography analyses (Fig. 3) and with epidemiological studies showing strong local spatial structure of the Omicron BA.1 wave (14, 15). In a supplementary analysis, we included booster vaccine uptake (per capita at the LTLA level) as a predictor under a time-inhomogeneous model, but we did not find it to be significantly supported (supplementary material), possibly due to collinearity with other predictors (peak timing in case incidence and case-sample residuals) or due to limited spatial heterogeneity in vaccine uptake.

## Discussion

We find that a substantial proportion of SARS-CoV-2 infections during the Omicron BA.1 wave in England can be traced back to a small number of introductions inferred to have occurred before or during the early travel restrictions on incoming passengers from southern Africa. Although the rate of importation continued to increase after mid-December, local onward transmission was observed only for a proportion (~25%) of imports that arrived after Christmas 2021. These results augment previous investigations of VOCs in England and other countries (30, 41), highlighting that the impact of international travel restrictions is limited if applied after local exponential growth is established and in the absence of local control measures. Here we conclude that the epidemics of BA.1 in multiple locations outside the country of first detection substantially contributed to the exponential growth of BA.1 importation into England in December 2021 (28). Thus, the practical effect of targeted travel restrictions can be constrained by the existence of multiple pathways between any two countries in the global aviation network, often via highly-connected locations with large travel volumes that can act as early secondary sources (39). UK travel restrictions were intended to delay the rapid expansion of BA.1 locally while offering additional vaccination to at-risk individuals. However, it is likely that Omicron had already spread internationally by the time it was detected in late November 2021, allowing secondary locations of VOC export to become established (28, 42). Therefore any proposed global systems that intend to rapidly detect and respond to new VOCs (and emerging infectious diseases in general) need to be designed around the connection structure of human mobility networks. Despite this, there are likely to be scenarios under which travel restriction can help control, contain, or delay the spread of emerging infections (43, 44) and much further theoretical and empirical work is needed to improve and inform rapid decision making concerning travel during public health emergencies.

Our continuous and discrete phylogeographic analyses (Fig. 3 & 4) jointly show how Omicron BA.1 disseminated rapidly across England, with Greater London playing a central role in its initial dissemination. Early viral movements outside of Greater London were dominated by medium-to long-distance travel from there; local transmission chains in recipient locations were observed later, coinciding with an increase in human mobility after the winter holidays (Fig. S18). The epidemic is revealed to be a network-driven phenomenon with an initial expansion phase that is well described by a gravity model, followed by a period of sustained local transmission propagated by local human mobility (39).

With this study, we can now compare the transmission histories of three successive VOC waves in England (Alpha (16), Delta (17), and Omicron) and contrast the factors that influenced their dispersals. First, Omicron and Delta were both introduced through international importation, whereas Alpha appeared to have originated in England (45). For both Omicron and Delta, early introductions from their presumed location of origin were followed by an increase in importation intensity from secondary locations. However, early transmission clusters for Delta were observed mainly in North West England, whereas most early Omicron infections were found in Greater London (15, 20). Second, different local NPIs and restrictions on within-country travel were implemented during the VOC waves. Although the introduction of Delta occurred during a period of relaxed NPIs, initial spreading was delayed due to a lower level of mobility following a national lockdown (17), whereas Omicron was introduced when human mobility had mostly recovered to a pre-pandemic level (Fig. S18). For the Alpha wave, rapid expansion from the South East was observed as a result of holiday travels (16) and was subsequently brought under control when NPIs were introduced, leading to reduced levels of local mobility (16). Third, spatial variations in population immunity from prior infections are likely to have impacted the dissemination of each VOC differently. For example, we expect the spread of Delta to be relatively unaffected by population immunity due to widespread infections and vaccination, and similarly for Omicron due to the antigenic novelty of the variant (9, 46, 47); whereas the initial growth rates of Alpha were affected by local variations in previous attack rates (16). These findings highlight two key questions for future work: how do the spatiotemporal interactions between importation and local transmission shape the spread of an invading VOC, and how can we efficiently evaluate the interplay of factors that drive the dissemination of an emerging VOC within a country.

Findings from our phylodynamic analysis should be interpreted in the context of several limitations. First, as discussed previously (30), the number of importation events identified is likely to underestimate the true number of independent introductions due to incomplete sampling and uneven sequencing coverage worldwide (48). Nevertheless, we were able to cross-validate our phylogenetic results using independent epidemiological data (Fig. S7 and Fig. S8). Second, to maintain computational tractability and remove potential sampling bias in the phylogeographic reconstruction of local transmission lineages, we included only a subset (about 7%) of the available English SARS-CoV-2 genomes from COG-UK, while accounting for geographical variations in sequencing coverage and COVID-19 prevalence. Despite this subsampling procedure, we note that spatial and temporal sampling was not perfectly representational (Fig. 4A and Fig. S9). This could be caused by geographical variation in case reporting rate or because the maximum sequencing capacity was exceeded in locations with exceptionally high case incidence. Third, our phylogenetic GLM analysis that explores the association of factors with virus lineage movement should be interpreted in light of potential biases in the mobility data. For example, movements in sparsely populated locations may be poorly captured due to censoring to protect user anonymity, and the degree to which smartphone mobility data is representative of the whole population could be affected by variation in smartphone use among locations. Work is ongoing to assess the benefit of human mobility data in the prediction and description of infectious diseases invasion dynamics.

Omicron BA.1 was replaced by lineage BA.2 in February 2022 and later by lineage BA.5 in June 2022 (20, 21). While the public health burden of COVID-19 has lessened due to reduced average disease severity and increased population immunity, the continued antigenic evolution of SARS-CoV-2 means that future variants with increased virulence remain possible. One priority in preparing for the next SARS-CoV-2 variant or novel pathogen emergence is to develop and implement robust pipelines for large-scale genomic and epidemiological analyses supported by unified data infrastructures (49, 50) a challenging task that will be realised only through the coordination of public health efforts worldwide.

## Supplementary Material

1

## Data Availability

UK genome sequences used were generated by the COVID-19 Genomics UK consortium (COG-UK, https://www.cogconsortium.uk/). Data linking COG-IDs to location have been removed to protect privacy, however if you require this data please visit https://www.cogconsortium.uk/contact/ for information on accessing consortium-only data. The Google COVID-19 Aggregated Mobility Research Dataset used for this study is available with permission from Google LLC. Code to reproduce the analyses will be made available on our GitHub repository.
