# Supplementary material for: Genomic assessment of invasion dynamics of SARS-CoV-2 Omicron BA.1
Source: Science. Author manuscript; Available in PMC 2024 Feb 14. (PMC10866301; doi:10.1126/science.adg6605)
Supplement: 1 [file NIHMS1965075-supplement-1.pdf]

Science
AAAS

Supplementary Materials for

Genomic assessment of invasion dynamics of SARS-CoV-2 Omicron BA.1

Joseph L.-H. Tsui[1,]†*, John T. McCrone[2,3,]†, Ben Lambert[4,]†, Sumali Bajaj[1,]†, Rhys P.D. Inward[1,]†, Paolo Bosetti[5,]†, Houriiyah Tegally[6,7] , Verity Hill[3,8], Rosario Evans Pena[1], Alexander E. Zarebski[1], Thomas P. Peacock[9,10], Luyang Liu[11], Neo Wu[11], Megan Davis[12], Isaac I. Bogoch[13], Kamran Khan[12,13], Meaghan Kall[10], Nurin Iwani Binti Abdul Aziz[10], Rachel Colquhoun[3], Áine O'Toole[3], Ben Jackson[3], Abhishek Dasgupta[1], Eduan Wilkinson[12,13], Tulio de Oliveira[6,7], The COVID-19 Genomics UK (COG-UK) consortium⇑, Thomas R. Connor[14,15,16], Nicholas J. Loman[17], Vittoria Colizza[18], Christophe Fraser[19,20], Erik Volz[21], Xiang Ji[22], Bernardo Gutierrez[1], Meera Chand[10], Simon Dellicour[23,24], Simon Cauchemez[5,]‡, Jayna Raghwani[1,25,]‡, Marc A. Suchard[26,]‡, Philippe Lemey[24,]‡, Andrew Rambaut[3,]‡, Oliver G. Pybus[1,20,25,]‡*, Moritz U.G. Kraemer[1,20,]‡*

†contributed equally as first authors
‡contributed equally as senior authors
⇑Consortium members and affiliations are listed in the Supplementary Material.
*Corresponding authors: lok.tsui@new.ox.ac.uk, oliver.pybus@biology.ox.ac.uk and moritz.kraemer@biology.ox.ac.uk

The PDF file includes:

Materials and Methods
Figs. S1 - S18
Tables S1 - S4
References

Materials and Methods

Genomic data

All SARS-CoV-2 sequences used in this study were downloaded on 12 April 2022. All available international (non-England, including Wales, Scotland and Northern Ireland independently) sequences were downloaded from GISAID (*25*) while English samples marked as community surveillance (pillar 2) were acquired from COG-UK. Historically, pillar 2 testing sites were instructed to select a number of 96 well plates for sequencing proportional to the fraction of total tests that week. Pillar 2 surveillance is intended to represent a random sample of community cases in the UK, with only 8% of being associated with testing for special reasons, i.e. 'attended-event', 'attended-outbreak-venue', 'confirmatory-test-borders', 'contact-testing-study', 'test-for-contact-self-referral', 'test-for-contact-tracing', 'test-for-contact-tracing-app', 'venue-outbreak'. However, given the changes in testing behaviour and regulations that occurred during the study period we can not rule out the possibility that there are some biases in the data set. These were partially addressed in the subsampling mentioned below.

Sequences were aligned and filtered as part of the COG-UK datapipe analysis hosted by CLIMB. This analysis removed duplicate and environmental sequences, and flagged samples with improbable collection dates (see https://github.com/COG-UK/datapipe for details). All sequences with impossible or improbable collection dates were removed. To further minimise dating errors caused by retrospective sequencing, only samples published to COG-UK or GISAID (*25*) within four weeks of sample collection were included. Sequences were aligned to the reference Wuhan-Hu-1 (genbank accession MN908947.3) with minimap2 and samples with less than 93% coverage were discarded. Sequence coverage weights were calculated for English sequences (https://github.com/robj411/sequencing_coverage) to ensure they could be subsampled proportional to the number of reported cases in each Upper Tier Local Authority using a two week sliding window. Scorpio was run as part of Pangolin and sequences identified as BA.1 or BA.2 were selected for further analysis.

Variant and Mutations (VAM) line list (from UK Health Security Agency)

The variant and mutations (VAM) line list compiled and provided by the UK Health Security Agency (UKHSA) contains epidemiological metadata of specimens sequenced from the pillar 2 mass testing programme by the COVID-19 Genomics UK Consortium (COG-UK). From the variant line list we extracted the traveller status (Traveller, Contact of Traveller, Not Travel-associated, Refused or Uncontactable, Awaiting Information) and specimen date of all sampled individuals who were tested positive for Omicron B.1.1.529 (BA.1) between 1 November 2021 and 31 January 2022.

Estimated Omicron BA.1 case incidence (from COVID-19 case count and S-gene target failure data)

Daily number of new COVID-19 cases by specimen date in each LTLA were downloaded from https://coronavirus.data.gov.uk/details/download (last assessed on 26 June 2022). S-gene target failure data were provided by UKHSA via a data sharing agreement. The presence of a genetic deletion on the spike protein of the Omicron BA.1 sub-variant produces SGTG in most PCR tests which can be used as a proxy for BA.1 infections. We used daily SGTF PCR-positive tests as a proxy (because these were time- and cost-effective as a test compared to genetic sequencing to ascertain variants) for Omicron BA.1 infection in conjunction with reported case data to estimate daily number of new BA.1 cases. However, small sample sizes in the SGTF dataset could lead to extreme scaling, i.e. zero or 100% of cases could be attributed to BA.1 infections if for example none or all samples were SGTF positive. Hence we calculated BA.1 cases in a Bayesian framework using uninformative Beta(1, 1) priors and the observed proportion of BA.1 infections (from the SGTF dataset) to estimate the

posterior proportion of BA.1 cases which was then scaled up by the number of reported cases from the coronavirus data download. We can also use the estimated uncertainty from the posterior distribution to get lower and upper bounds in the scaled up BA.1 case numbers.

International passenger flight data arriving in England

We evaluated travel data generated from the International Air Transport Association (IATA) to quantify passenger volumes originating from international airports and arriving in England. IATA data accounts for approximately 90% of passenger travel itineraries on commercial flights, excluding transportation via unscheduled charter flights (the remainder is modelled using market intelligence).

Estimated importation intensity of Omicron BA.1 from potential exporters

We estimated and compared the weekly importation intensity of SARS-CoV-2 Omicron BA.1 from 27 countries (including Scotland and Northern Ireland independently) with the highest air passenger volumes arriving in England between 7 Nov 2021 and 26 March 2022 (collectively accounting for ~80% of the total air passenger volume during this period). The weekly importation intensity is an estimate of the number of Omicron BA.1 cases imported during a given week from a specified source location, calculated by multiplying together the estimated weekly relative prevalence of Omicron BA.1 at the source location and the number of air passengers arriving from the source location.

We estimated the weekly number of air passengers arriving in England using monthly air traffic data, assuming a uniform daily distribution of passengers throughout the month and aggregating to a weekly level. To account for potential biases that might result from differences in case reporting rate, we used test positivity as a proxy for the underlying weekly prevalence at the source locations. Daily test positivity rates at the country level were downloaded from OWID (https://ourworldindata.org/; last accessed on 3 April 2023) and their weekly averages were computed. For Scotland and Northern Ireland, daily test positivity rates were downloaded from GOV.UK COVID-19 Dashboard (https://coronavirus.data.gov.uk/; last accessed on 23 April 2023). To account for local (within-country) heterogeneities in Omicron BA.1 prevalence and air traffic volume, we calculated EIIs from Spain and the United States at the autonomous community- and state-level. Weekly test positivity rates for autonomous communities in Spain were downloaded from the European Centre for Disease Prevention and Control Data Dashboard (https://www.ecdc.europa.eu/en/publications-data/archive-historical-data-testing-volume-covid-19; last assessed on 22 April 2023). For the US, weekly test positivity rates at the state level were calculated using data from https://github.com/govex/COVID-19/blob/master/data_tables/testing_data/time_series_covid19_US.csv (last assessed on 4 April 2023). We note that three different approaches were used to calculate test positivity for the US states depending on the availability of different test statistics. The three different approaches are: (A) positive specimens / total specimens, i.e. the number of positive PCR tests divided by the total number of PCR tests given, (B) positive people / total encounters, i.e. number of people who tested positive (PCR) divided by the total number of PCR tests given, and (C) positive people / total specimens, i.e. the number of people who tested positive (PCR) divided by the total number of PCR tests given. For states where multiple measures of the positivity rates are possible, the optimal approach was applied according to the order (A), (B) and (C), with approach (A) being the optimal approach. For Washington state (WA) in particular, no appropriate measure of the denominator in the calculation of positivity rate is available using any of the approaches, and as a result the national average positivity rates were used instead. We also note that no reliable testing data for Egypt can be found; Egypt is therefore omitted in this EII analysis and only included in subsequent sensitivity analysis as detailed below.

In a sensitivity analysis, we separately calculated EIIs using weekly COVID-19 case incidence per capita as a proxy for prevalence at the source locations (Fig. S6). Data sources are the same as in the analysis using test positivity rates (with the inclusion of Egypt using data from OWID (https://ourworldindata.org/; last accessed on 3 April 2023). Similarly, EIIs for Spain and the US were calculated at the autonomous community- and state-level. To highlight the potential bias that might have resulted from variations in case reporting rate between countries, the weekly number of tests performed per capita is calculated for each country (Fig. S4)

UK population estimates

Mid-year population estimates for England in 2020 at the LTLA level were downloaded from https://www.ons.gov.uk/peoplepopulationandcommunity/populationandmigration/populationestimates/datasets/populationestimatesforukenglandandwalesscotlandandnorthernireland. Population sizes were used as the denominator in calculating numbers of COVID-19 cases per capita and normalised local mobility in each LTLA.

Vaccination data with age breakdown

Daily vaccination data with age breakdown at the lower tier local authority (LTLA) level were downloaded from https://coronavirus.data.gov.uk/metrics/doc/vaccinationsAgeDemographics. The dataset consists of daily cumulative number and percentages of people who have received either a 1st dose, 2nd dose, or booster dose (of any type) since the start of the pandemic in each LTLA, with age breakdown by roughly 5-year intervals (5-11, 12-15, 16-17, 18-24, 25-29, 30-34, 35-39, 40-44, 45-49, 50-54, 55-59, 60-64, 65-69, 70-74, 75-79, 80-84, 85-89, 90+).

Aggregated and anonymised human mobility data

We used the Google COVID-19 Aggregated Mobility Research Dataset described in detail in (*51*, *52*), which contains anonymized relative mobility flows aggregated over users who have turned on the *Location History* setting, which is turned off by default. This is similar to the data used to show how busy certain types of places are in Google Maps — helping identify when a local business tends to be the most crowded. The mobility flux is aggregated per week, between pairs of approximately 5km^2 cells worldwide, and for the purpose of this study further aggregated for LTLAs in the United Kingdom (https://geoportal.statistics.gov.uk/datasets/lower-tier-local-authority-to-upper-tier-local-authority-december-2016-lookup-in-england-and-wales/explore) for the time period of November 2019 to January 31st, 2022.

To produce this dataset, machine learning is applied to log data to automatically segment it into semantic trips. To provide strong privacy guarantees (*53*), all trips were anonymized and aggregated using a differentially private mechanism to aggregate flows over time (see https://policies.google.com/technologies/anonymization). This research is done on the resulting heavily aggregated and differentially private data. No individual user data was ever manually inspected, only heavily aggregated flows of large populations were handled. All anonymized trips are processed in aggregate to extract their origin and destination location and time. For example, if n users travelled from location a to location b within time interval t, the corresponding cell (a,b,t) in the tensor would be n∓err, where err is Laplacian noise. The automated Laplace mechanism adds random noise drawn from a zero mean Laplacian distribution and yields (□, δ)-differential privacy guarantee of □ = 0.66 and δ = 2.1 × 10−29 per metric. Specifically, for each week W and each location pair (A,B), we compute the number of unique users who took a trip from location A to location B during week W. To each of these metrics, we add Laplace noise from a zero-mean distribution of scale 1/0.66. We then remove all metrics for which the noisy number of users is lower than 100, following

the process described in (*53*), and publish the rest. This yields that each metric we publish satisfies (ε,δ)-differential privacy with values defined above. The parameter □ controls the noise intensity in terms of its variance, while δ represents the deviation from pure □-privacy. The closer they are to zero, the stronger the privacy guarantees.

These results should be interpreted in light of several important limitations. First, the Google mobility data is limited to smartphone users who have opted in to Google's *Location History* feature, which is off by default. These data may not be representative of the population as whole, and furthermore their representativeness may vary by location. Importantly, these limited data are only viewed through the lens of differential privacy algorithms, specifically designed to protect user anonymity and obscure fine detail. Moreover, comparisons across rather than within locations are only descriptive since these regions can differ in substantial ways.

Changes in case reporting rate in the United Kingdom

To assess the degree of changes in case reporting rate in the United Kingdom, we compared the weekly national case incidence downloaded from the GOV.UK COVID-19 Dashboard with that estimated by the UK Office of National Statistics (ONS). Specifically, since we were interested in the relative changes over time rather than the absolute values, a linear regression of the ONS case incidence estimates against case incidence from the GOV.UK COVID-19 Dashboard was performed and the residuals from the model were examined (Fig. S10).

As described in further details below, Omicron sequences from England were subsampled with sample weights calculated from the ratio between the cumulative number of reported cases and the cumulative number of sequences collected in the preceding two weeks for any given date. Therefore, to assess the potential bias that might have resulted from changes in case reporting rate in the context of the subsampling of English genomes, a similar linear regression analysis as above was performed, with additional (two-preceding-weeks) smoothing applied to both the ONS and GOV.UK case incidences (Fig. S10).

Phylogenetic analysis and importation analysis

We developed a large-scale phylogenetic analysis pipeline following a similar approach as in du Plessis et al. (2021) (*54*) with additional extensions and modifications to ensure the computational tractability of analyses of up to hundreds of thousands of SARS-CoV-2 sequences (*55*) (Fig. S1).

First, the study period was divided into two phases: (1) from 21 November 2021 (sample date of the earliest known genome of the Omicron variant in England, sequenced retrospectively) to 28 November 2021, and (2) from 29 November 2021 to 31 January 2022. The time of division between the two phases was chosen on the basis of an expected change in importation intensity as a result of the implementation of travel restrictions targeted at multiple southern African countries starting on 28 November 2021. With the relatively few genomes available from the first phase and to account for an increased risk of importations prior to the travel restrictions, all 874 available sequences (from both England and non-England locations) were included. Owing to the large number of genome samples collected during the second phase, a downsampling strategy was applied to ensure that the analysis was computationally tractable. To generate a manageable dataset of global sequences, first we computed a crude estimate of the number of new Omicron cases in each country in each epi-week by multiplying the number of reported COVID-19 cases (downloaded from https://github.com/owid; last accessed on 4 May 2022) by the proportion of sampled genomes that were of the Omicron variant PANGO lineages BA.1 and BA.2, using metadata available from GISAID (*25*) (https://gisaid.org/;

last accessed on 12 April 2022). The number of global sequences to be sampled in each epi-week was then allocated in proportion to the estimated total number of Omicron lineages BA.1 and BA.2 cases in the week whilst maintaining a dataset size of ~50,000. In a given epi-week, countries with an estimated number of Omicron cases that accounted for at least 0.5% of the estimated global total were considered as potential exporters. Genome samples were then allocated in proportion to the estimated number of cases among these potential exporters, with the remaining allocation randomly distributed among the non-exporter countries. There was a slight enrichment for samples collected in the early phase of the Omicron wave (early December 2021), where we ensured that a minimum of 4,000 genomes were sampled for each epi-week where available. A similar approach was used to curate a dataset of 21,039 Omicron genomes sampled from Wales, Scotland and Northern Ireland, again using relevant metadata from GISAID (*25*) and epidemiological data available on (https://api.coronavirus.data.gov.uk/v1/data; last accessed on last accessed on 4 May 2022). This downsampling procedure resulted in a dataset of 59,647 global (non-English) sequences. To generate a dataset of English genomes of roughly the same size, 60,000 sequences were randomly sampled from the COG-UK master alignment whilst accounting for variations in sequencing coverage and prevalence amongst UTLAs over time, using the same method as in Volz et al. (*56*). This resulted in a combined dataset of 140,686 genomes of which 42.6% were sampled in England with the remaining from non-England locations.

Despite substantial downsampling, estimating a phylogenetic tree for hundreds of thousands of SARS-CoV-2 sequences remains a challenge, with most standard programs only able to handle up to thousands of sequences. To tackle this, we first estimated a maximum likelihood (ML) tree for the 874 sequences collected during the first phase of the study period using IQTREE (*57*) with the GTR+G substitution model, rooted with reference genome Wuhan-Hu-1 (GenBank accession MN908947.3) as an outgroup. Five molecular clock outliers were identified and subsequently removed, after examining the root-to-tip regression plot from TreeTime (*58*). The resulting tree was then used as a starting tree from which a parsimony tree was estimated by inserting individual sequences sequentially and in chronological order according to sample dates, using the recently developed UShER placement tool (*59*). During each step in the iterative process, all sequences sampled on a given date were considered for placement whilst excluding sequences with 5 or more equally parsimonious placements. Sequences excluded in a previous step were appended to the next batch for reconsideration. The resulting tree was then optimised through 6 iterations of matOptimize (*60*) with SPR radius of 40 and 100 for the first 5 and final iteration respectively. This iterative tree building process resulted in a phylogeny of 115,634 sequences (with 25,921 (18.3%) sequences excluded due to uncertainty in sample placement). Next we used Chronumental (*61*) (a recently developed time-tree estimation tool for handling large phylogenies) to estimate a randomly resolved time-calibrated tree, with inferred tip dates that maximise the evidence lower bound under a probabilistic model. By comparing the inferred tip dates with sample dates and examining a root-to-tip plot, 12 molecular clock outliers were further removed, resulting in a final phylogeny of 115,622 sequences.

To further reduce the computational resources and time required, we divided the phylogeny estimated above into smaller tree partitions according to sub-lineage (of Omicron) assignment as defined by the Pango nomenclature (*62*). Using a custom Python script, subtrees with a high degree of clustering of sequences of the same descendant lineage of Omicron were identified, whilst accounting for some level of ambiguity in lineage assignment (e.g. a tree partition may contain up to 25% of sequences that are of a minority sub-lineage before it is subdivided into multiple partitions), as would be expected given the high sampling density and variations in sequencing quality. Further merging of these

identified subtrees resulted in five final tree partitions, labelled BA.1 (n=38,522), BA.1.1 (n=37,028), BA.1.15 (n=12,229), BA.1.17 (n=21,549), and BA.2 (6,294) according to the sub-lineage represented by the majority of sequences in each partition. Given that the primary focus of this study is the invasion dynamics of Omicron BA.1 in England, the BA.2 partition was omitted in all further downstream analyses.

Having divided the phylogeny into smaller tree partitions of computationally manageable size, we then performed time-calibration of the subtrees using a recently implemented model in BEAST v1.10 (*63*) which replaces the traditional tree-likelihood with a more efficient likelihood based on a simple Poisson model, thus allowing Bayesian phylogenetic analyses of up to tens of thousands of sequences. In this approach, the tree operators are constrained such that only node heights and polytomy resolutions are sampled, whilst the tree topology is fixed to that of a data tree which we generated using Treetime (*58*) with a fixed clock rate of 7.5x10E-4 substitutions/site/yr. Using a Skygrid coalescent tree prior (*64*) with grid points every two weeks, we ran between 2 and 6 MCMC chains of 3×10^8 to 2.4×10^9 iterations for each tree partition independently. The first 33% to 40% of each chain was discarded as burn-in and resampled every 1×10^6 to 2.4×10^8 states before merging using LogCombiner, resulting in 1,200 posterior tree samples for each tree partition. Model convergence and mixing was assessed using Tracer (*65*).

To reconstruct the importation dynamics of Omicron BA.1, we then used a two-state asymmetric discrete trait analysis (DTA) model implemented in BEAST v1.10 (*63*), using the posterior tree samples estimated above as the empirical tree distributions. For each tree partition, we ran two MCMC chains of 5 million iterations each, resampled every 9,000 states and with the first 10% discarded as burn-in. TreeAnnotator 1.10 (*63*) was used to generate a maximum clade credibility (MCC) tree for each subtree, in which each internal node is assigned a posterior probability of representing a transmission event in England. Nodes with a posterior probability of >0.5 were identified as introductions; a small number of nodes with ambiguous location assignment (posterior probability = 0.5) were ignored in downstream analyses. To identify the local transmission lineage resulting from each of the introductions, a depth-first search was performed following the same procedure as in du Plessis et al. (2021) (*54*), where a path starting from each internal node that corresponds to an introduction is traversed forwards in time until a non-England node is encountered or there are no more nodes to be explored. By convention, introductions that led to only a single sampled English sequence were labelled as singletons; only introductions that led to more than one observed local transmission event were labelled as transmission lineages. The time of importation of each transmission lineage was estimated by taking the mid-point between the internal node corresponding to the introduction and its parent.

Our methodology estimating the time of importation of transmission lineages is likely to result in an apparent “expansion” of the temporal profile of inferred importation intensity (daily number of infected travellers arriving in England) relative to its true underlying distribution. This could be explained by an increase in importation lag (time elapsed between when a lineage is inferred to have been imported and the first detected local transmission event) over time as shown previously by du Plessis et. al. (*30*), due to transmission lineages from later importations having fewer genomes as they have less time to grow, and are therefore less likely to be captured by genomic surveillance assuming a constant sampling intensity. To verify this effect, we compared the inferred importation intensity from the above phylodynamic analysis with empirical observations from testing data recording international travellers, extracted from the Variant Mutation line list provided by UKHSA (Fig. S2).

However, we note that the robustness of this comparison is potentially limited by variations in sampling intensity in the epidemiological data as a result of rapidly-changing testing policies for arriving travellers in the United Kingdom during the latter part of January 2022 (*66*).

Exponential growth of daily frequency of importations

In the absence of any travel restrictions and changes in human mobility as a result of the emergence of a new VOC, the importation intensity during the initial phase of the invasion would be expected to follow a pattern of exponential growth that mirrors the increase in number of infections in the exporting countries. To verify and examine any potential deviation from this pattern, we fitted a simple exponential model to the 7-day rolling average daily number of importations inferred from the phylodynamic analysis. Specifically, we fitted the model using least-squares regression to the inferred daily numbers of importations during the period between the beginning of November 2021 and a range of cut-off dates. The cut-off date that resulted in the highest adjusted R^2 value can be interpreted as an estimate of the time when the growth of importation intensity began to deviate from an exponential trajectory.

Continuous phylogeographic analysis

To reconstruct the spatiotemporal patterns of the Omicron BA.1 wave in England, all local transmission lineages (as identified from the MCC trees generated from the 2-state discrete trait analysis above) with five or more sequences were extracted for continuous phylogeographic analyses. Each sequence was assigned a latitude and a longitude randomly from within the postal district (metadata provided by COG-UK) where the sample was collected. For each transmission lineage, we performed the continuous phylogeographic reconstruction on a fixed (pruned from the MCC tree) using a relaxed random walk model (*67*) implemented in BEAST 1.10.4 (*63*), with a Cauchy distribution to model the among-branch heterogeneity in dispersal velocity. Following a similar approach as in McCrone et al. (*17*), the eight largest transmission lineages (labelled A to H, from largest to smallest) containing >700 sequences were inferred independently, with the remaining smaller transmission lineages (n=524) inferred in a single joint analysis with a shared diffusion model (i.e. same parameter estimates for likelihood, precision matrix, correlation, etc, but independent estimates for diffusion rate and trait likelihood). Owing to variations in the extent of spatial dispersal among these smaller transmission lineages (with larger lineages being more spatially dispersed in general), 30 were subsequently removed from the joint analysis and inferred independently. Model convergence and mixing was assessed using Tracer v1.7 (*65*). For the independent analyses of the eight largest transmission lineages, we ran between 2 and 5 MCMC chains of 200 to 300 million iterations, sampling every 10,000 to 80,000 states and removing the first 10% to 33% of each chain as burn-in, resulting in 10,000 to 13,5000 trees sampled from the posterior distribution. For the independent analyses of the 30 smaller transmission lineages with fewer than 700 sequences, we ran 2 MCMC chains each of 200 million iterations which we then merged after resampling every 30,000 states and removing the first 10% as burn-in, giving 12,000 posterior trees per transmission lineage. Finally, in the joint analysis, 8 independent chains of 200 million were run with sampling every 120,000 states. They were combined after removing the first 10% as burn-in, again resulting in 12,000 posterior tree samples for each transmission lineage. These posterior tree samples were then used to generate an annotated MCC tree for each transmission lineage using TreeAnnotator (*63*).

To facilitate subsequent analyses of viral lineage movements at the LTLA level, we mapped the inferred location of each internal node in the transmission lineages to its corresponding LTLA by checking whether the inferred coordinates are contained within the associated polygon. In the case where an enclosing polygon could not be found (e.g. a small proportion of internal nodes were

inferred to lie in the small spaces between neighbouring polygons), the polygon that is geographically closest to the inferred location was assigned.

Discrete phylogeography with generalised linear model (GLM) parameterisation

We used the approach of discrete phylogeography with generalised linear model (GLM) to parameterise transition rates between locations and test the association of viral lineage dispersal with a number of geographical, demographic, epidemiological and human mobility-related factors (see Table S2 for full list of predictors). Specifically, to test the gravity model as a predictor of viral lineage movements, we considered in the GLM analysis the population size at the origin and destination location of each movement and the geographical distance between them. To further capture any heterogeneities in aggregated human mobility at the city-level (which are unlikely to be adequately described by the gravity model), we also included the aggregated mobility matrix and community memberships as predictors. We allowed these mobility-related predictors to vary across different phases in the time-inhomogeneous model to test for temporal variations in aggregated human mobility patterns and also potentially time-varying effect of mobility on viral dispersal. We observed from both epidemiological data and continuous phylogeography that many LTLAs in Greater London experienced an earlier uptick in Omicron BA.1 cases compared to most LTLAs with other regions of England. To capture this asynchronicity in local epidemic dynamics and investigate its impact on viral dispersal, we considered in the GLM analysis whether each viral movement started or ended in the Greater London region and additionally the time of first peak in Omicron BA.1 case incidence at the origin and destination location. Furthermore, we also tested for the impact of sampling bias by including a predictor based on the residuals from a simple regression of sample size against Omicron BA.1 cases for both the origin and destination location. Due to the small number of sequences collected in some LTLAs especially during later phases of the epidemic, the regression residuals were computed using sample sizes and case counts aggregated over the whole study period in both the time-homogeneous and time-inhomogeneous models.

Unlike continuous phylogeography where each sequence is assigned a unique set of coordinates in continuous space, discrete phylogeography requires that sequences are grouped into discrete geographical units. The level of granularity of these geographical units depends on a number of factors including (i) the desired level of resolution at which the dispersal history is to be reconstructed, (ii) the amount of heterogeneities present within each geographical unit, and (iii) the maximum number of geographical units beyond which the analysis becomes computationally intractable. To capture heterogeneities in viral movements at the city-level and to allow comparisons with results from continuous phylogeography, we allocated sequences to their corresponding LTLAs using a lookup table which provides unique mapping between postal districts and LTLAs.

The current computational architecture and implementation of the discrete phytogeographic GLM model limits the number of discrete units possible to 256, which is smaller than the number of LTLAs across which sequences were sampled for some of the larger transmission lineages. To tackle this, we aggregated LTLAs where appropriate to reduce the number of geographic units. In order to minimise the resulting information loss, we first considered LTLAs with the fewest sequences and performed a merging operation if an adjacent LTLA with at least one sampled genome could be found. In the case where multiple adjacent LTLAs were available, the LTLA with the most number of sampled genomes was chosen for the merger. After each merging operation, the list of LTLAs (or geographical units after merging) ranked by the number of sampled genomes was recalculated for the next iterative step (it is therefore possible for an LTLA to be involved with multiple merging operations). This process continued until there were only 253 geographic units in each transmission lineage. For the

geographical units consisting of multiple LTLAs, each statistic of interest was averaged over the relevant LTLAs, weighted by population size where appropriate. For transmission lineages with sequences sampled in fewer than 256 LTLAs, no merging was performed.

The discrete phylogeographic GLM model parameterizes the log of between-location transition rates as a log linear function of the predictors. Continuous predictors (geographical distances, population sizes, aggregated mobility matrices, peak timing in case incidence, sampling residuals) were therefore log-transformed and standardised after adding a pseudo-count to each entry where appropriate. Binary variables (community memberships, Greater London/non-Greater London) were encoded as 0 and 1. In the mobility-related predictors, there was missing data for one or two geographical units in some transmission lineages (due to mobility data being unavailable for South Tyneside and City of London), which we labelled as NA and later integrated out in our Bayesian inference. For the aggregated mobility matrix predictor with continuous values in the large-scale transmission analyses, we confronted this using a new Hamiltonian Monte Carlo (HMC) kernel to jointly sample all missing covariates from their posterior distributions building on similar efforts in the BEAST framework (*68*, *69*). The HMC kernel produces distant proposals with relatively high acceptance rate for the Metropolis algorithm by exploiting numerical solutions to the Hamiltonian dynamics. We performed the analyses using the code available in the hmc-clock branch of the BEAST codebase (available at https://github.com/beast-dev/beast-mcmc/tree/hmc-clock) in conjunction with the BEAGLE code available in the hmc-clock branch of the codebase (available at https://github.com/beagle-dev/beagle-lib/tree/hmc-clock). We ran the analyses on a set of 100 empirical trees for each transmission lineage extracted from the BEAST importation analysis and ran sufficiently long chains sampling every 500 generations, or combined multiple chains (excluding adequate burn-ins), to ensure effective sample sizes (ESSs) > 100 for the continuous parameters as diagnosed using Tracer (*65*). A custom R script was used to summarise and visualise the posterior coefficient estimates and inclusion probabilities of each predictor.

Discrete phylogeography with GLM: effect of booster uptake

The rollout of booster vaccination in the United Kingdom began in September 2021 (*70*) and was initially prioritised for those aged 50 and above as they are at a higher risk of severe symptoms and hospitalisation from infection. Eligibility for boosters was extended to those aged 40 and above on 22 November 2021 (*71*), and subsequently to all adults on 30 November 2021 (*72*). This resulted in spatial variations in booster uptake that are strongly correlated with the underlying age structure of the population (Fig. S16, A and C), which is in turn correlated with Omicron BA.1 prevalences due age-dependent transmission patterns as shown by Elliott et al. (2022) (*15*) (Fig. S16, B and D).

To adjust for age structure as a confounder, here we devise an effective measure of the booster uptake that is independent of the underlying age structure of the population. Using vaccination data (downloaded from the GOV.UK COVID-19 Dashboard) consisting of the percentage of people in different age groups who have received a booster dose, we calculate the overall booster uptake in each Lower Tier Local Authority (LTLA) assuming an age distribution that is the same as the national population-weighted average age distribution (computed from mid-2020 population estimates published by the UK Office of National Statistics). This is equivalent to the overall proportion of the population who would have received a booster dose in an LTLA given its observed age-specific booster uptake (with roughly 5-year grouping), assuming that it has the same age structure as the national average. Similar to other covariates included in the GLM analysis, for the geographical units consisting of multiple LTLAs, the effective booster uptake is averaged over the relevant LTLAs weighted by population size.

Discrete phylogeography with GLM: likelihood-deviance measure

To evaluate the relative importance of the different predictors in the time-inhomogeneous GLM analysis, we have developed and implemented a new phylogeographic model-fit measure which builds upon standard, permutation-based machine learning approaches to assessing variable importance (*73*).

Starting from the posterior $\square(\square|\square, \square_{11}, \ldots, \square_{\square\square}, \ldots, \square_{\square 2})$ with phylogeographic likelihood $\square(\square|\square, \square_{11}, \ldots, \square_{\square\square}, \ldots, \square_{\square 2})$ where Θ represents all model parameters and $\square_{\square\square}$ represents the vector of covariate values for covariate $\square \in \{1, \ldots, \square\}$ ($\square$ being the total number of predictors in the model) in epoch $\square \in \{1,2\}$, we define the deviance for this covariate as $\square_{\square\square} = \square\square\square\ \square(\square|\square, \square^{\square}_{11}, \ldots, \square^{\square}_{\square\square}, \ldots, \square^{\square}_{\square 2}) - \square\square\square\ \square(\square|\square, \square_{11}, \ldots, \square_{\square\square}, \ldots, \square_{\square 2})$ where $\square^{\square}_{\square\square}$ is a random permutation of the observed covariate vector $\square_{\square\square}$. To estimate the posterior distribution of $\square_{\square\square}$, we computed values of $\square_{\square\square}$ for each MCMC sample, $\square$, with realised model parameters $\Theta^{(\square)}$ by setting $\Theta = \square^{(\square)}$, and randomly permuting $\square_{\square\square}$ with equal probability for all possible permutations. For each covariate and in each epoch, we estimated the posterior distribution of the likelihood-deviance resulting from a random permutation of the covariate values. We then compared the resulting posterior distributions and ranked the importance of each covariate in predicting the geographic locations $\square$. The covariate marginal posterior modal (most probable) ranking was then reported, as shown in Fig. 4 and Fig. S14 (with 1 being most important). While this approach averages over all possible marginal permutations and therefore has improved performance over earlier permutation-based measures in machine learning (*74*), it may nevertheless return limited discrimination among highly correlated covariates. Permute-and-relearn importance methods (*75*) are able to overcome this limitation but remain computationally impractical given the numbers of tips and the size of the state-spaces considered in this study.

Using the above approach, we find that the predictor rankings do not always reflect differences in absolute effect size and that they help to identify similarities and differences between transmission lineages, as well as between epochs for a given transmission lineage. In the two largest transmission lineages, the gravity model covariates are consistently the most important covariates, in both the early- and late-epoch. For Transmission Lineage-A, the Greater London origin predictor is the next important predictor throughout the study period. The Greater London origin predictor is also more important in Transmission Lineage-A than in Transmission Lineage-B, for which a change in importance of this predictor between the early- and late-epoch is observed. In Transmission Lineage-B, the origin peak time predictor is the next important predictor after the gravity model predictors. For both transmission lineages, we observe a large and consistent increase in the importance of the mobility matrix predictor between the early- and late-epoch.

We also note that the magnitude of the likelihood-deviance estimates scales with the size of the dataset (and therefore the number of tips in the transmission lineages). As such, the deviance estimates do not provide a relative measure of fit across transmission lineages.

Branching process model and comparison of transmission lineage size distributions

To verify that the time of importation is the key determinant of transmission lineage size, we compared the size distribution of empirically observed transmission lineages with that from a model that simulates the branching process of transmission lineages following importation. Simulated importation dates are set to the dates estimated from the phylodynamic analysis and simulated

transmissions occur at the spatially homogeneous growth rates estimated from the daily number of reported COVID-19 cases (from the GOV.UK COVID-19 Dashboard) and SGTF data in England. Due to the low number of Omicron BA.1 cases at the beginning of the epidemic, which can lead to unreliable estimates of the initial growth rate, we performed a series of simulations with a range of different starting growth rates (taken from estimates during early parts of the invasion). We computed the Kullback-Leibler (KL) distance between the size distribution of simulated lineages and that of lineages inferred from phylodynamic analysis (Table S1). The growth rate that minimised the KL distance was then used to impute the initial growth rate in the best-fit model. We note that, given the simple nature of the model, we did not take into account any uncertainties associated with the case growth rates but relied only on the central estimates. As a sensitivity analysis for the potential bias that might have resulted from this and also any changes in case reporting rate during the study period, we repeated the simulations using case incidence estimates from the UK Office of National Statistics (ONS) (see Fig. S10 for comparisons between case incidence data from UK.GOV COVID-19 Dashboard and ONS estimates). We observed consistent results as those obtained using the case incidence data from the GOV.UK COVID19 Dashboard (Fig. S7 and Fig. S8).

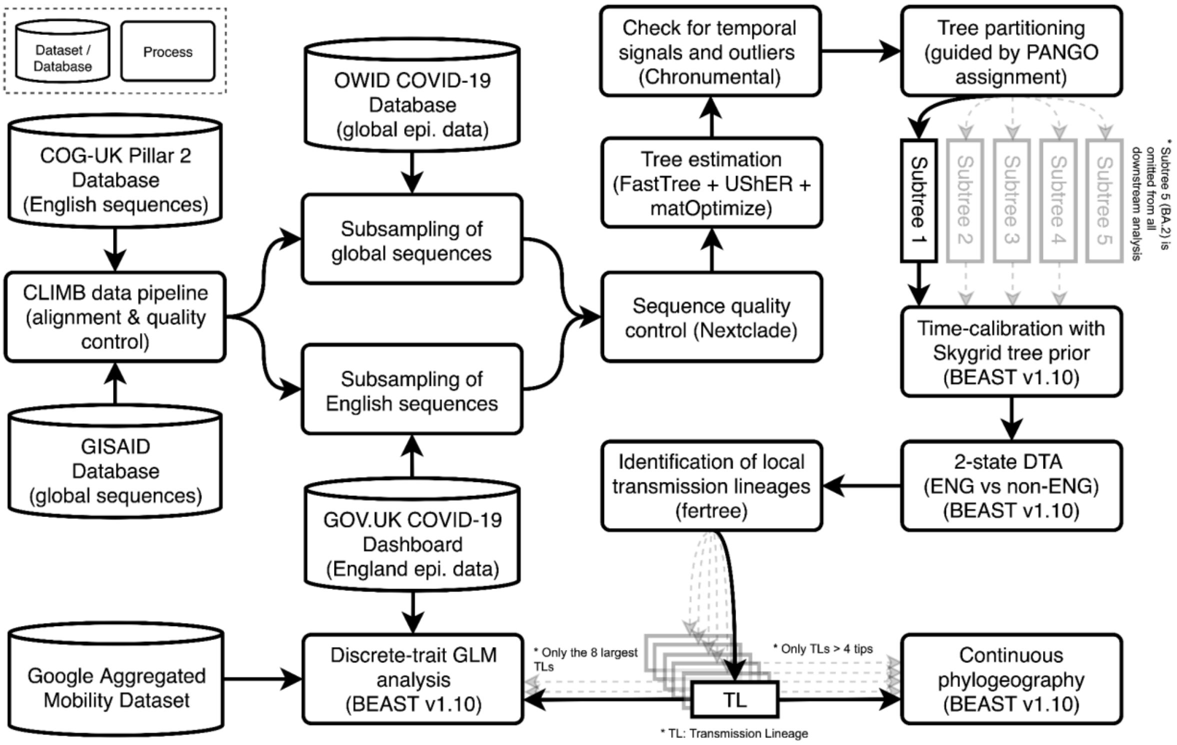

Fig. S1: Outline of phylogenetic analysis pipeline. *A high-level overview of the various processes and phylogenetic analyses performed, as well as any relevant programs and packages for each step. Note that each subtree (except for the subtree containing only Omicron BA.2 sequences which we have omitted from further downstream analysis) from the tree-partitioning procedure is passed onto further analysis independently. Please refer to materials and methods for a more detailed description of each analysis.*

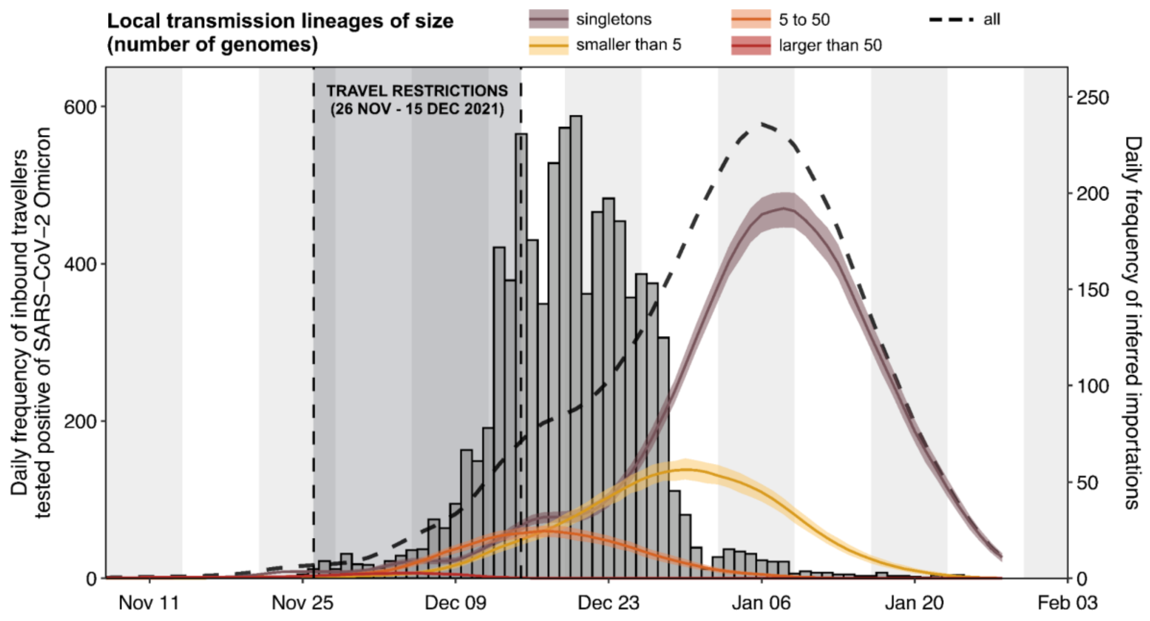

Fig. S2*: *Comparison of Omicron BA.1 importation intensity as observed empirically from Variant Mutation (VAM) line list versus estimates from phylodynamic analysis. *Histogram (grey bars) shows the daily number of incoming travellers who were later tested positive for Omicron BA.1 under the UKHSA mass testing programme (by specimen date). Solid lines represent the daily frequency of importations (7-day rolling average) as inferred from the phylodynamic analysis, coloured according to the size of resulting local transmission lineages (with the black dashed line representing the total numbers irrespective of size); shading denotes the 95% HPD across the posterior tree distribution.*

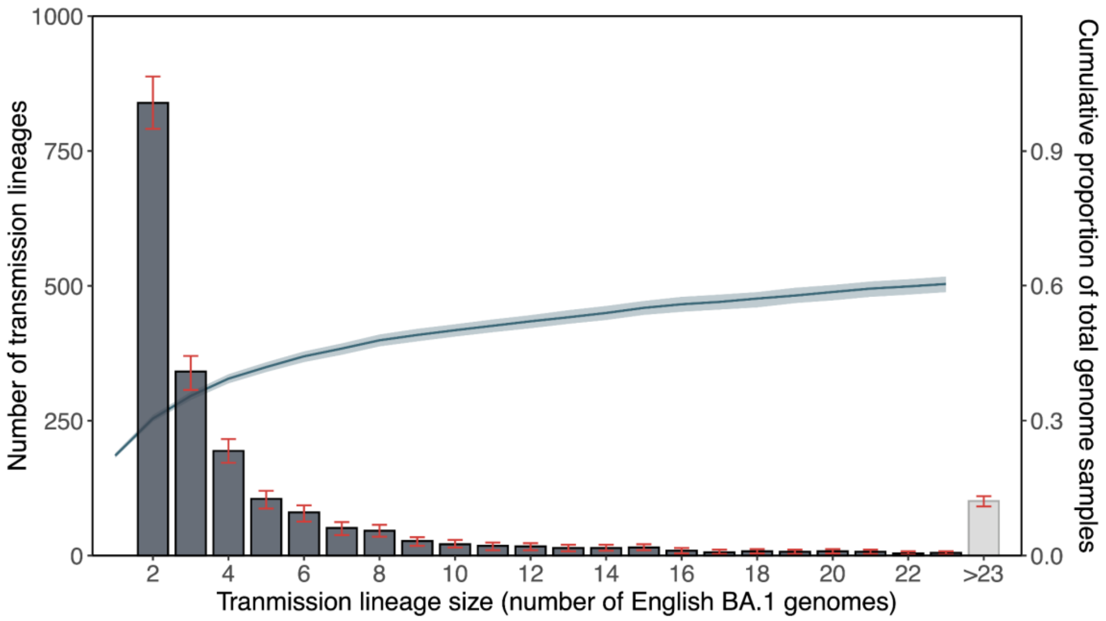

Fig. S3. Distribution of local transmission lineage sizes from phylodynamic analysis. *Grey bars show the number of transmission lineages of different sizes; red error bars denote the 95% HPDs across the posterior tree distribution. Blue solid line represents the cumulative proportion of English Omicron BA.1 genomes in our dataset accounted for by transmission lineages up to a certain size; shading denotes the 95% HPD across the posterior tree distribution.*

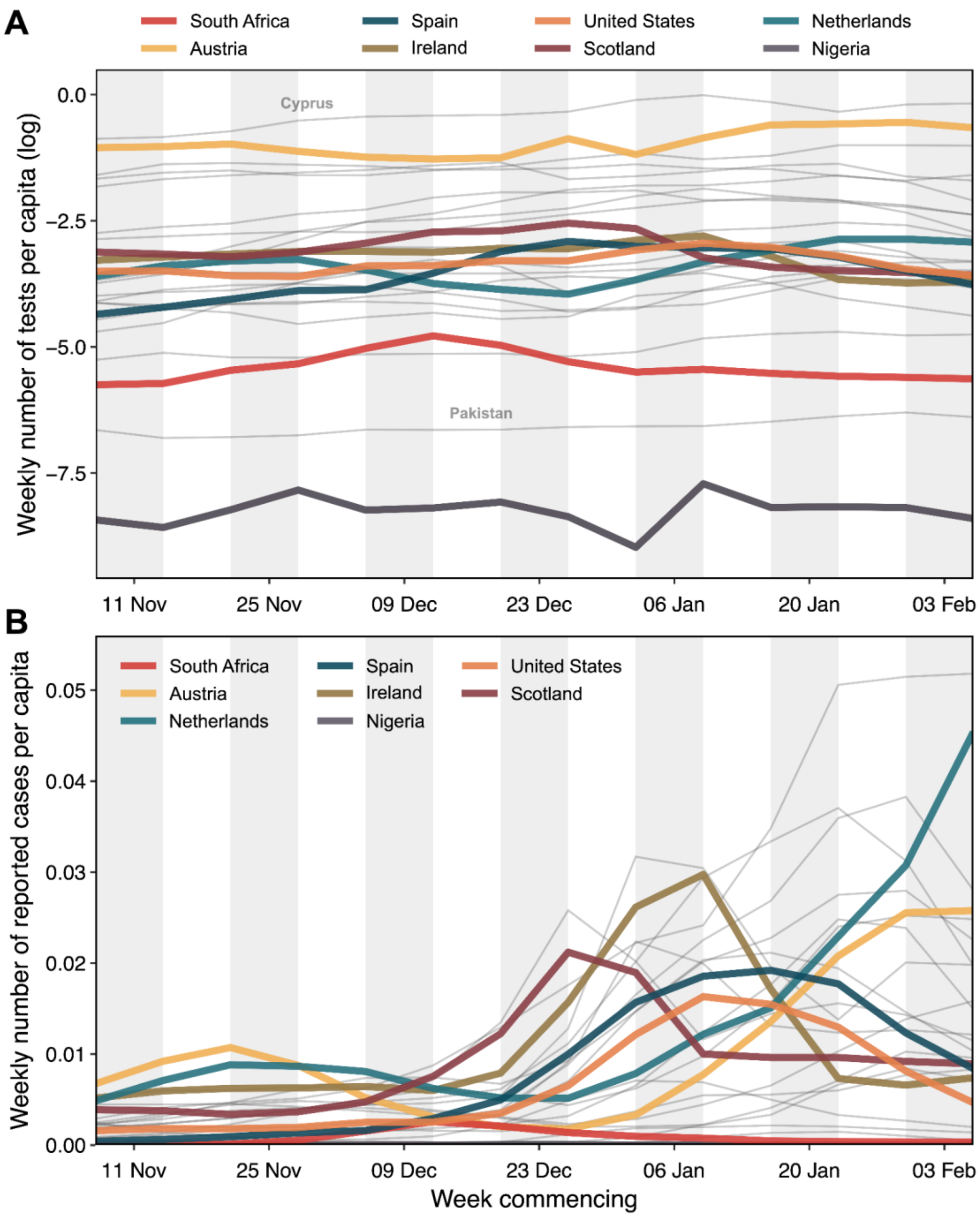

Fig. S4: Variations in case reporting rates between countries. *(A) Weekly number of tests performed per capita (log-transformed) and (B) weekly number of reported cases per capita for 27 countries (including Scotland and Northern Ireland independently) with the highest air passenger volumes arriving in England between November 2021 and January 2022 (collectively accounting for ~80% of total air passenger volume in this period). Thick solid lines represent a subset of eight selected countries with notable contribution to the overall intensity of Omicron BA.1 importation into England at different points during the study period; thin grey lines represent all other countries.*

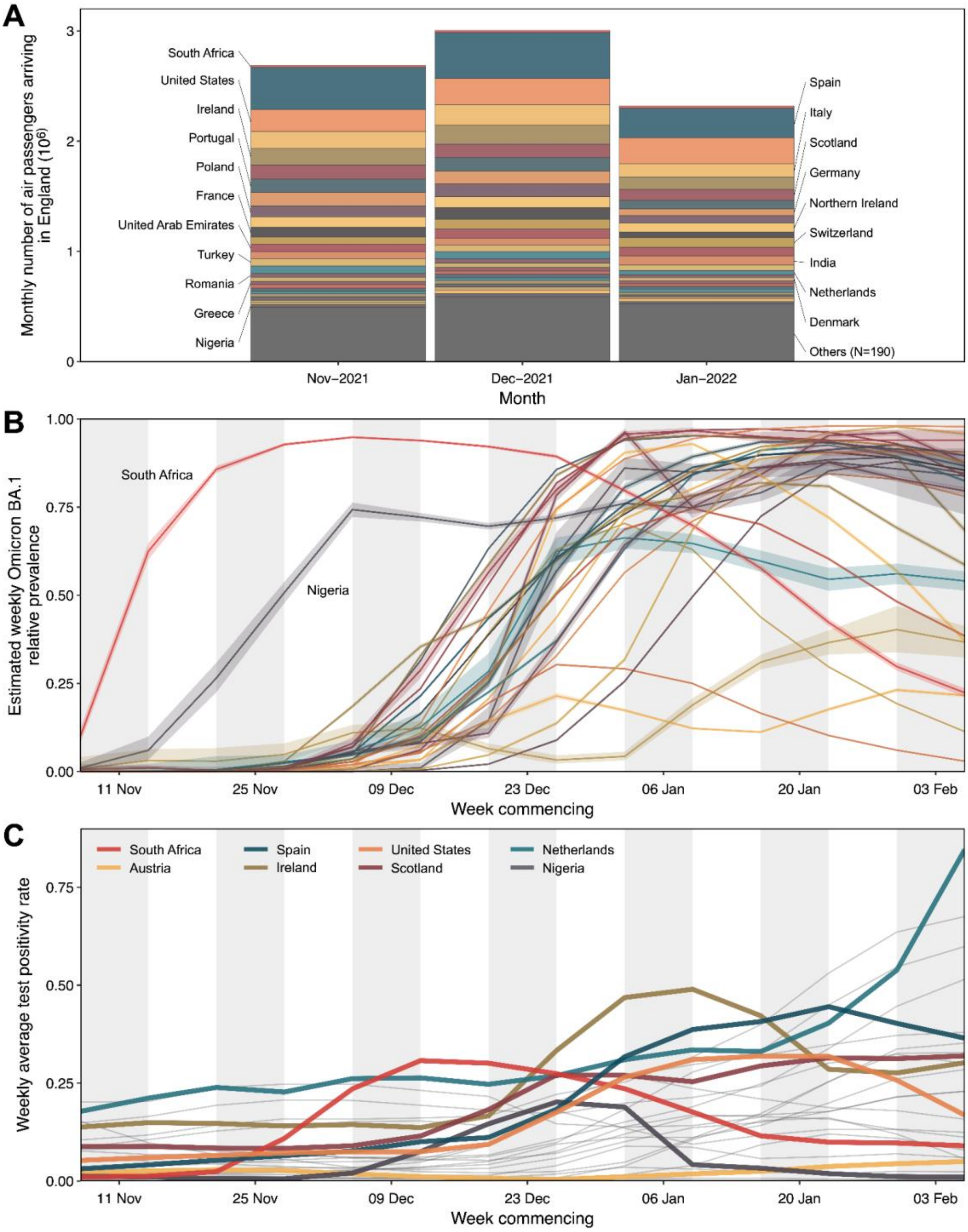

Fig. S5: Components of Estimated Importation Intensity (EII). *(A) Monthly number of air passengers arriving in England from all countries (N=217) between November 2021 and January 2022. Area of each coloured block indicates the number of air passengers arriving from a given country (out of the 27 countries for which EIIs are calculated) during a given month; grey blocks at the bottom represent air traffic volume from all other countries (N=190). (B) Estimated weekly relative prevalence of Omicron BA.1 in the 27 selected countries during the study period; shaded region represents the 95% CI. (C) Weekly average test positivity rate in the 27 selected countries during the study period. Thick solid lines represent a subset of eight selected countries with notable contribution to the overall intensity of Omicron BA.1 importation into England at different points during the study period; thin grey lines represent all other countries.*

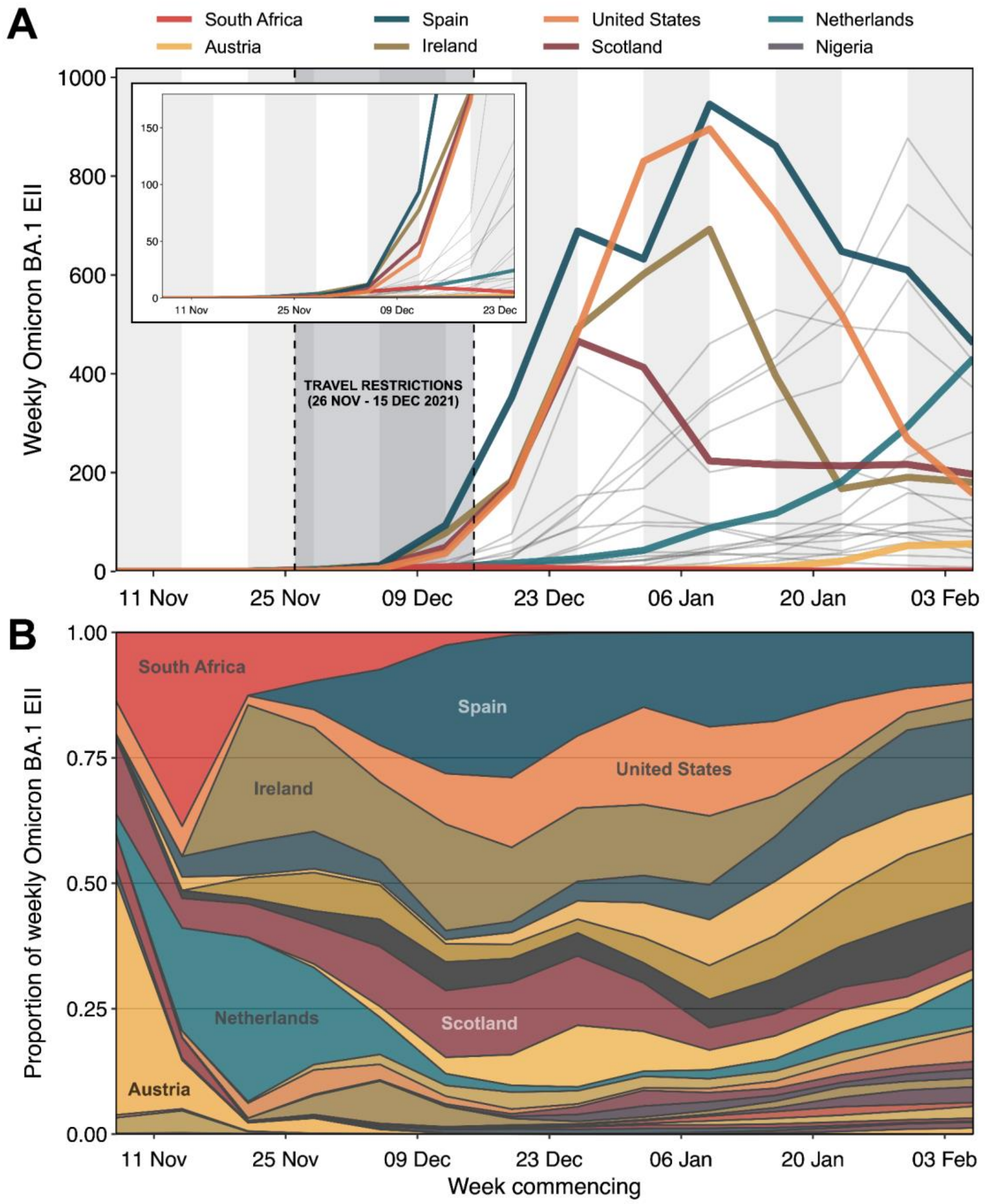

Fig. S6: Estimated Importation Intensity (EII) of Omicron BA.1 from selected potential exporters, using case incidence per capita as proxy for underlying prevalence. *Estimated weekly number of Omicron BA.1 cases arriving in England from 27 countries (including Scotland and Northern Ireland independently) with the highest air passenger volumes arriving in England between November 2021 and January 2022 (collectively accounting for ~80% of total air passenger volume in this period), using weekly number of reported cases as a proxy for trends in the underlying prevalence. Thick solid lines represent EIIs from eight selected countries with notable contribution to the overall intensity of Omicron BA.1 importation into England at different points during the study period; thin grey lines represent all other countries. Inset shows a magnified view of early trends. Grey shaded region represents the period (26 November to 15 December 2021) when travel restrictions on international arrivals from multiple southern African countries were implemented. (B) Relative proportion of weekly EII of Omicron BA.1 by location/country among selected potential exporters. Areas representing countries highlighted in (A) are labelled.*

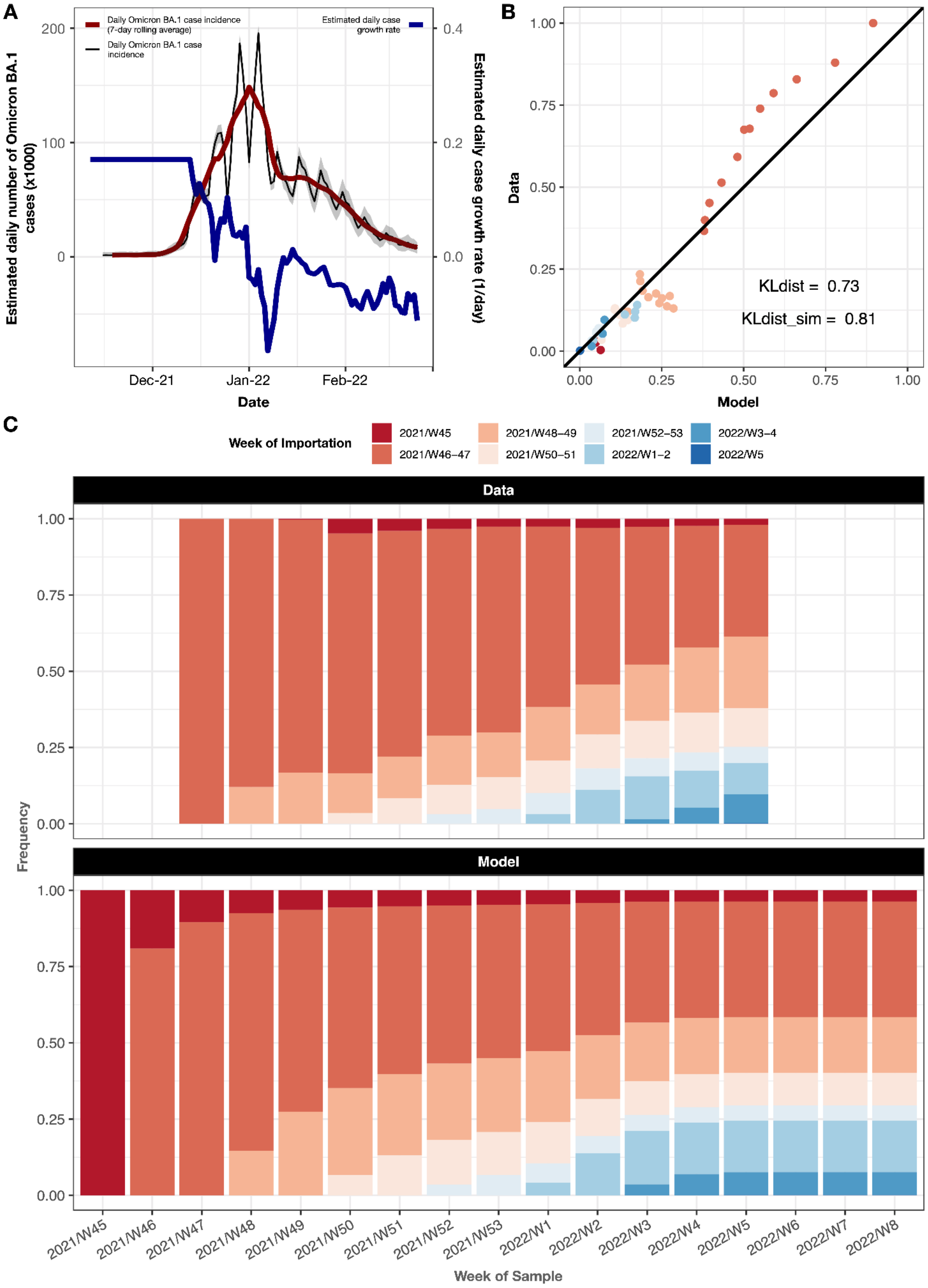

Fig. S7. Comparison of transmission lineage size distribution from phylodynamic analysis versus simulated results from a branching process model. *(A) Black and red solid lines represent the estimated daily and 7-day rolling average daily number of Omicron BA.1 cases in England. Grey*

shaded region represents the 95% CI associated with the estimated daily number of Omicron BA.1 cases. Blue solid line represents the estimated daily growth rate, with the initial values imputed using an estimate of the growth rate on 13 December 2021. (B and C) Weekly proportion of local Omicron BA.1 infections resulting from importations at different times throughout the epidemic, with comparison between empirical observations from the phylodynamic analysis (C, top) and predictions from the branching process model (C, bottom).

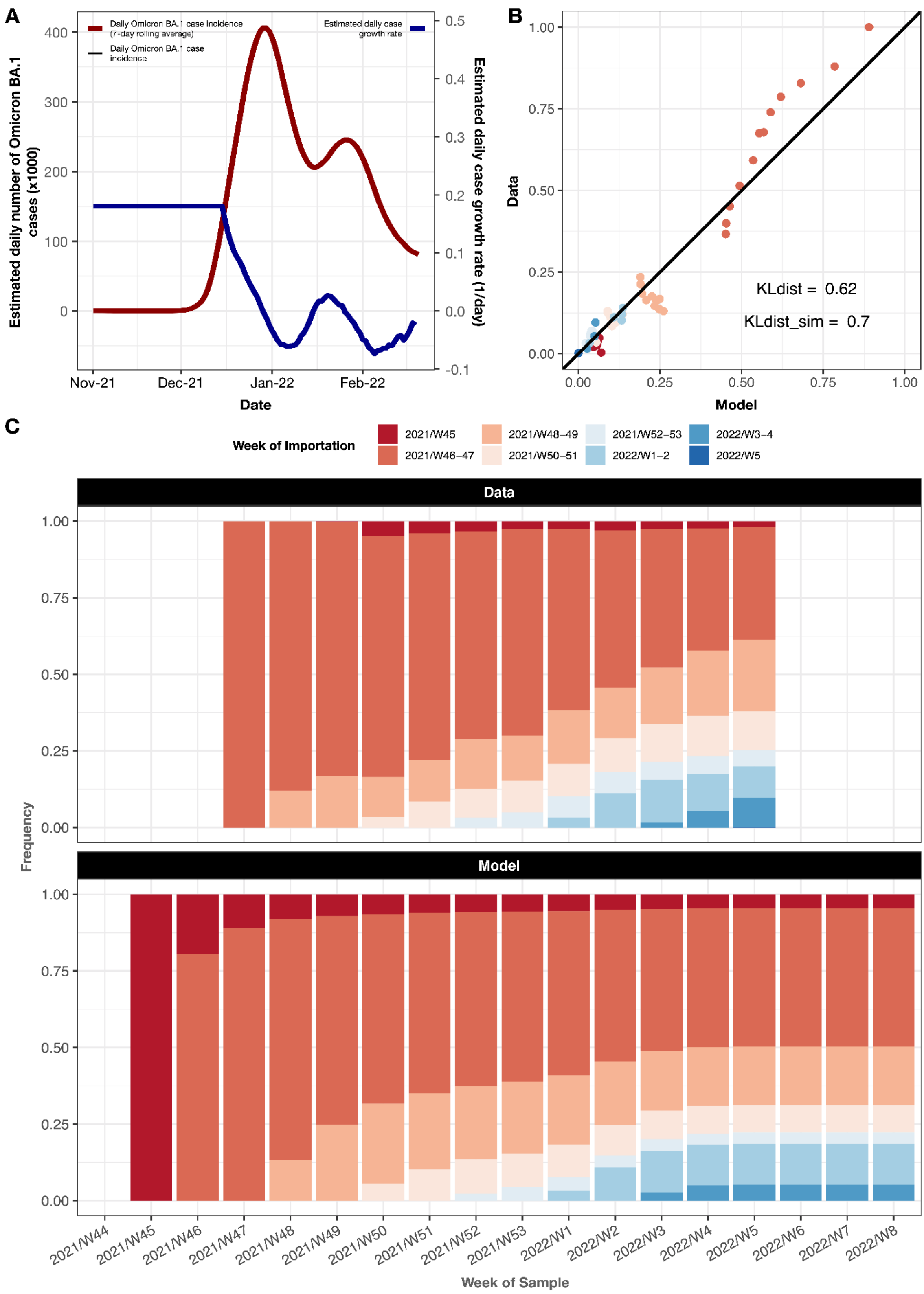

Fig. S8. Comparison of transmission lineage size distribution from phylodynamic analysis versus simulated results from a branching process model (sensitivity analysis using ONS case incidence estimates). *(A) Black and red solid lines represent the estimated daily and 7-day rolling average daily number of Omicron BA.1 cases in England, from the UK Office of National Statistics (ONS). Blue solid line represents the estimated daily growth rate, with the initial values imputed using an estimate of the growth rate on 13 December 2021. (B and C) Weekly proportion of local Omicron BA.1*

infections resulting from importations at different times throughout the epidemic, with comparison between empirical observations from the phylodynamic analysis (C, top) and predictions from the branching process model (C, bottom).

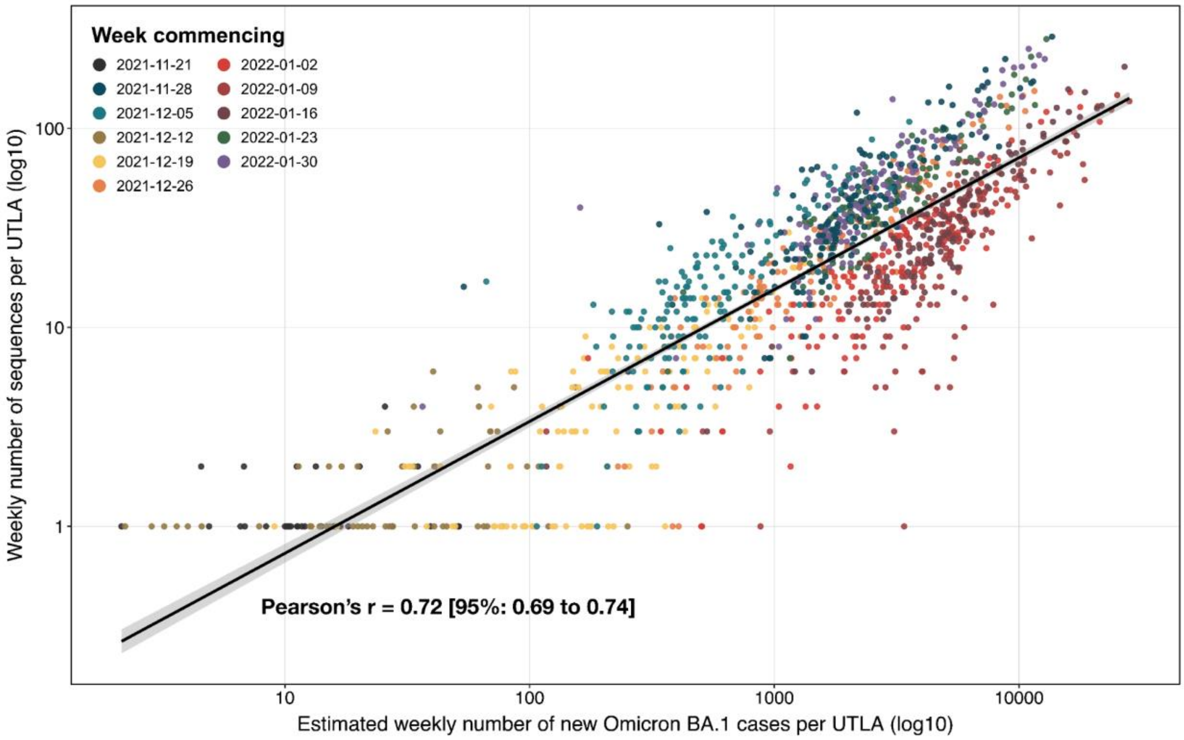

Fig. S9. Correlation between estimated number of Omicron BA.1 cases and number of Omicron BA.1 genomes sampled across UTLAs in England. *Circles are coloured by week commencing date. Solid black line represents the line of best-fit; shaded region represents the 95% CI. We note in particular the clustering of circles corresponding to the same week along the line of best-fit, indicating small changes in sequencing coverage across time but not across UTLAs.*

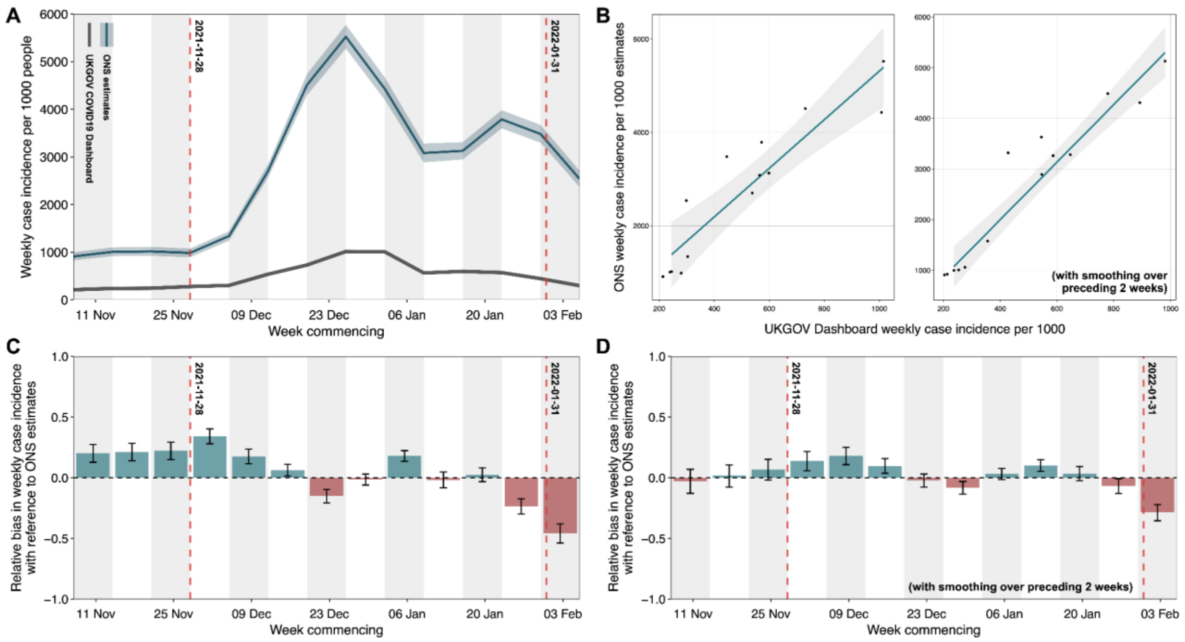

Fig. S10: Comparison of case incidence from the GOV.UK COVID19 Dashboard against estimates from UK Office of National Statistics. *(A) Weekly number of positive COVID-19 cases in England as reported by the GOV.UK COVID19 Dashboard (https://coronavirus.data.gov.uk/) (solid black line); weekly number of COVID-19 cases in England as estimated from positivity rates by the UK Office of National Statistics (ONS) (solid blue line; shading denotes the associated 95% CI). Vertical red dashed lines indicate the start date and end date of the period during which English genomes were sampled in proportional to weekly number of reported cases (see supplementary materials). (B) Weekly number of COVID-19 cases per 1000 people as estimated by ONS versus that from the GOV.UK COVID19 Dashboard, with (right) and without (left) smoothing over the preceding two weeks for each given date. Blue lines show the least-squares fit and the shading denotes the 95% CI. (C, D) Residuals from a linear regression between the weekly number of COVID-19 cases per capita as estimated by ONS versus that from the GOV.UK COVID19 Dashboard (during the period of interest from 28 November 2021 to 31 January 2022), with (D) and without (C) smoothing over the preceding two weeks for each given date. Error bars denote the 95% confidence interval associated with uncertainty in the ONS estimates; boxes are coloured red (negative) or blue (positive) according to the sign of the residuals.*

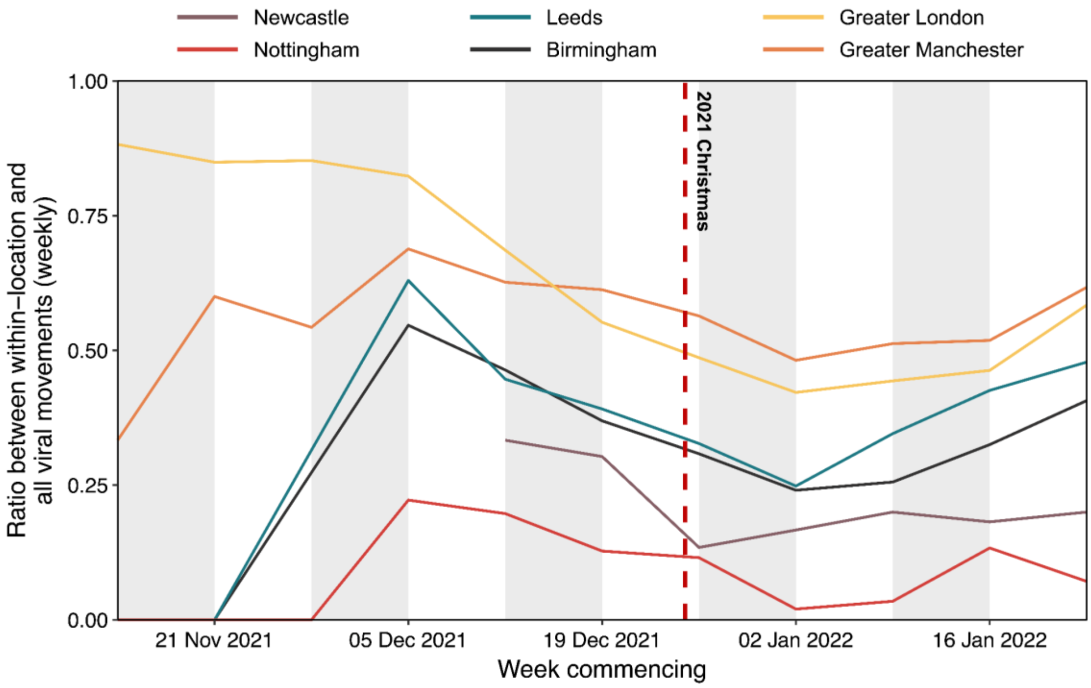

***Fig. S11. Within-location versus all viral lineage movements for major cities in England.** Each solid line represents the ratio between the frequency of within-location and all viral lineage movements per week, as inferred from continuous phylogeography for 6 major cities in England. For Greater Manchester and Greater London, viral lineages associated with multiple lower tier local authorities were aggregated in the calculation of these ratios. The timing of each viral lineage movement was assumed to be half-way between the inferred time of the nodes corresponding to the origin and destination.*

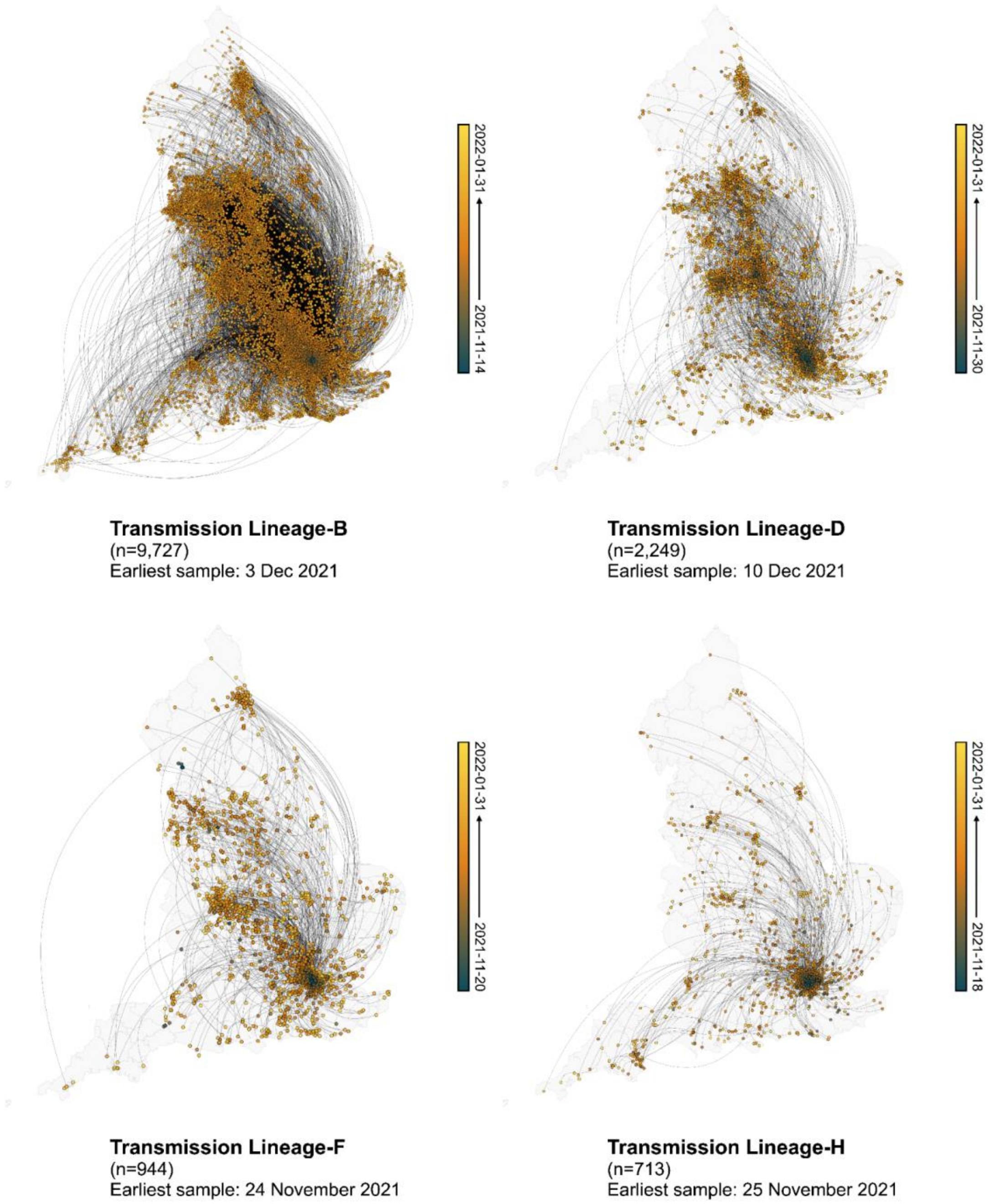

Fig. S12. Spatiotemporal dynamics of Omicron BA.1 transmission lineages in England (Transmission Lineage-B, D, F and H). *Maps showing viral lineage movements inferred from continuous phylogeography for Transmission Lineage-B, D, F and H. Nodes are coloured according to inferred date of occurrence and the direction of viral lineage movement is indicated by edge curvature (anti-clockwise).*

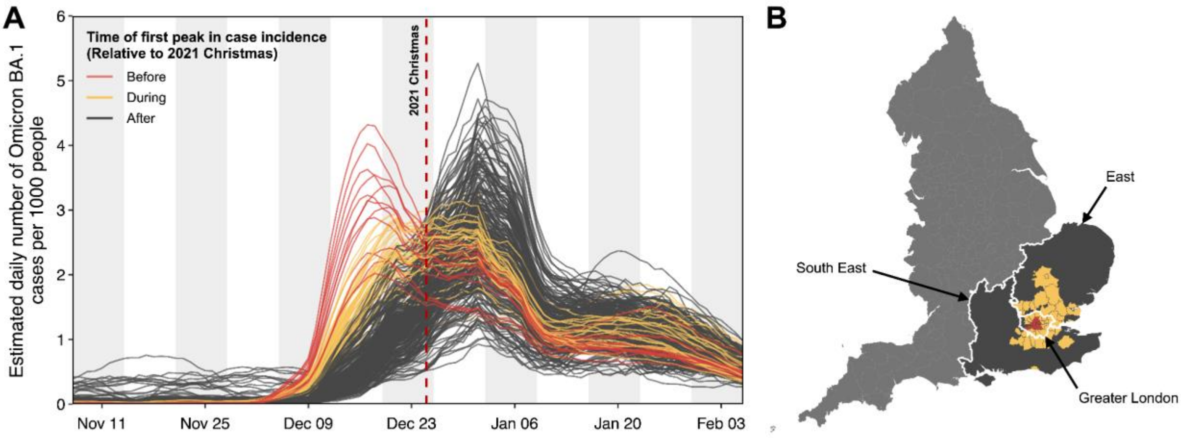

Fig. S13. Spatial variations in timing of first peak of Omicron BA.1 case incidence across England. *Estimated daily number of Omicron BA.1 cases per 1000 people at the Lower Tier Local Authority (LTLA) level (7-day rolling average), coloured according to the timing of their first peak relative to Christmas 2021 (specifically, whether the interval during which the daily number of Omicon BA.1 cases exceed 85% of the peak incidence lies entirely before (red), after(dark grey), or encloses (yellow) 25 December 2021 (Christmas). (B) Map showing the spatial distribution of the timing of the first peak in Omicron BA.1 case incidence at the LTLA level, following the same colour scheme as in (A).*

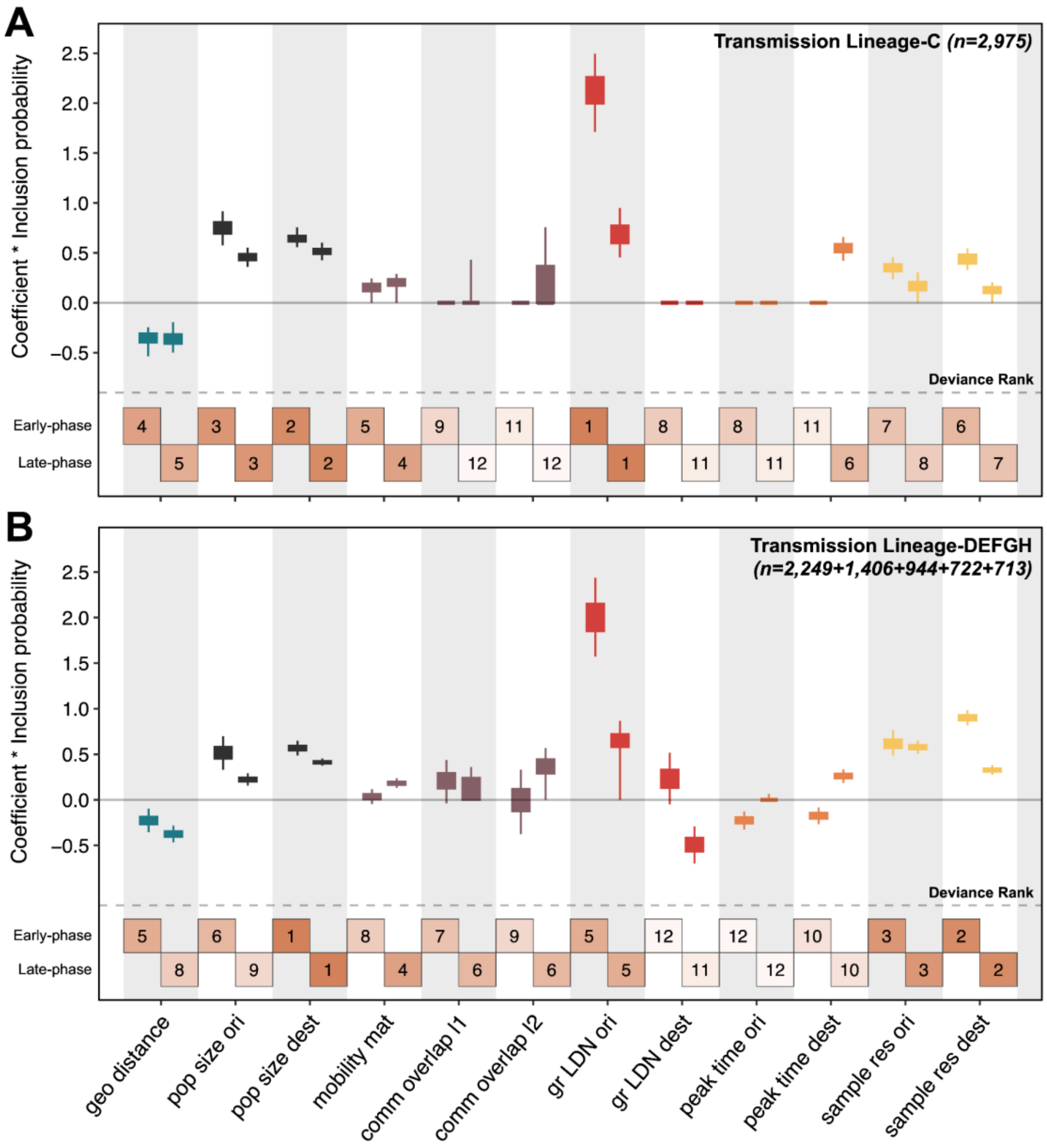

Fig. S14. Predictors of Omicron BA.1 viral lineage movements in England in the time-inhomogeneous discrete-phylogeography with GLM model. *For each predictor, the box and whiskers show the posterior distribution of the product of the log predictor coefficient and the predictor inclusion probability; the left hand value represents the expansion period estimate and the right hand value the post-expansion period estimate. Top panel (A) shows estimates for Transmission Lineage-C and bottom panel (B) shows those for Transmission Lineages D, E, F, G, and H analysed in a joint model. Posterior distributions are coloured according to predictor type: geographic distances (geo distance, dark blue), population sizes at origin and destination (pop size ori & pop size dest, black), aggregated mobility (mobility mat, purple), mobility-based community membership level 1 and level 2 (comm overlap l1 & l2, purple), Greater London origin and destination (gr LDN ori & gr LDN dest, red), time of peak incidence at the origin and destination (peak time ori & peak time dest, orange) and the residual of a regression of sample size against case count regression at either the origin and destination (sample res ori & sample res dest, yellow). Boxes at the bottom of each panel are numbered and shaded to represent the rank of predictors based on their deviance measure, with 1 indicating the largest (most important) and 12 indicating the smallest (least important).*

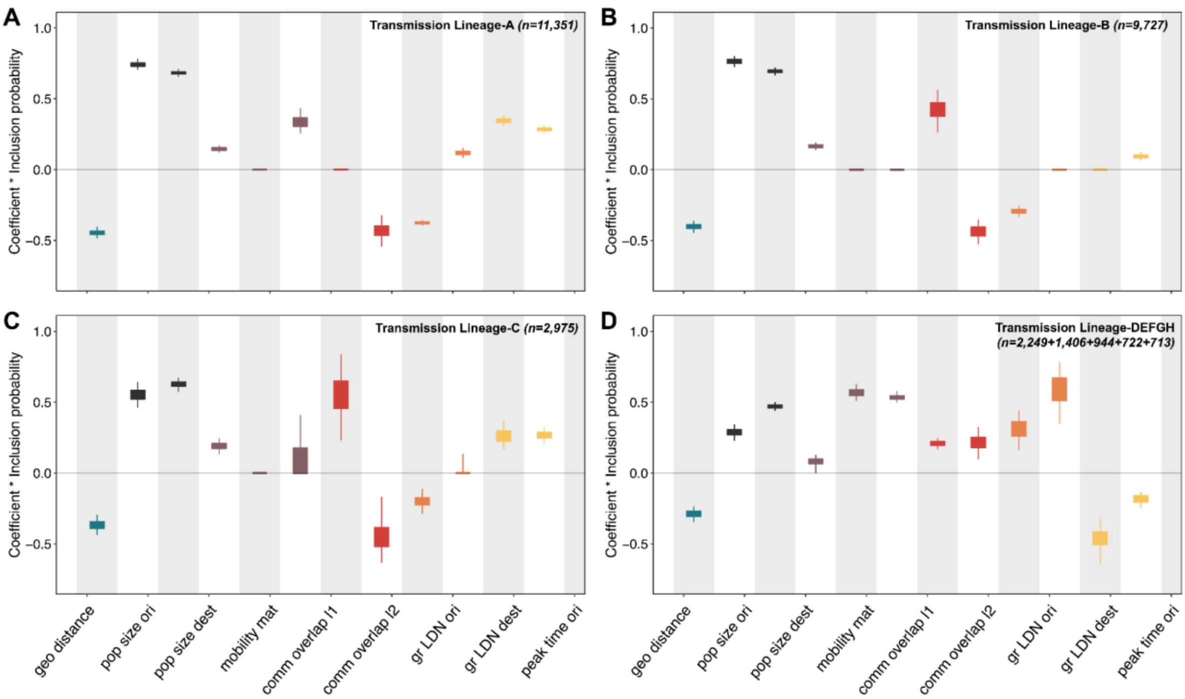

Fig. S15. Predictors of Omicron BA.1 viral lineage movements in England in the time-homogeneous discrete-phylogeography with GLM model. *Each panel corresponds to an independent analysis for Transmission Lineage-A (A), Transmission Lineage-B (B), Transmission Lineage-C (C), and Transmission Lineages D, E, F, G and H together in a joint model (D). For each predictor within a panel, the box and whiskers show the posterior distributions of the product of the log predictor coefficient and the predictor inclusion probability. Posterior distributions are coloured according to predictor type: geographic distances (geo distance, dark blue), population sizes at origin and destination (pop size ori & pop size dest, black), aggregated mobility (mobility mat, purple), mobility-based community membership level 1 and level 2 (comm overlap l1 & l2, purple), Greater London origin and destination (gr LDN ori & gr LDN dest, red), time of peak incidence at origin and destination (peak time ori & peak time dest, orange) and the residual of a regression of sample size against case count regression at either the origin and destination (sample res ori & sample res dest, yellow).*

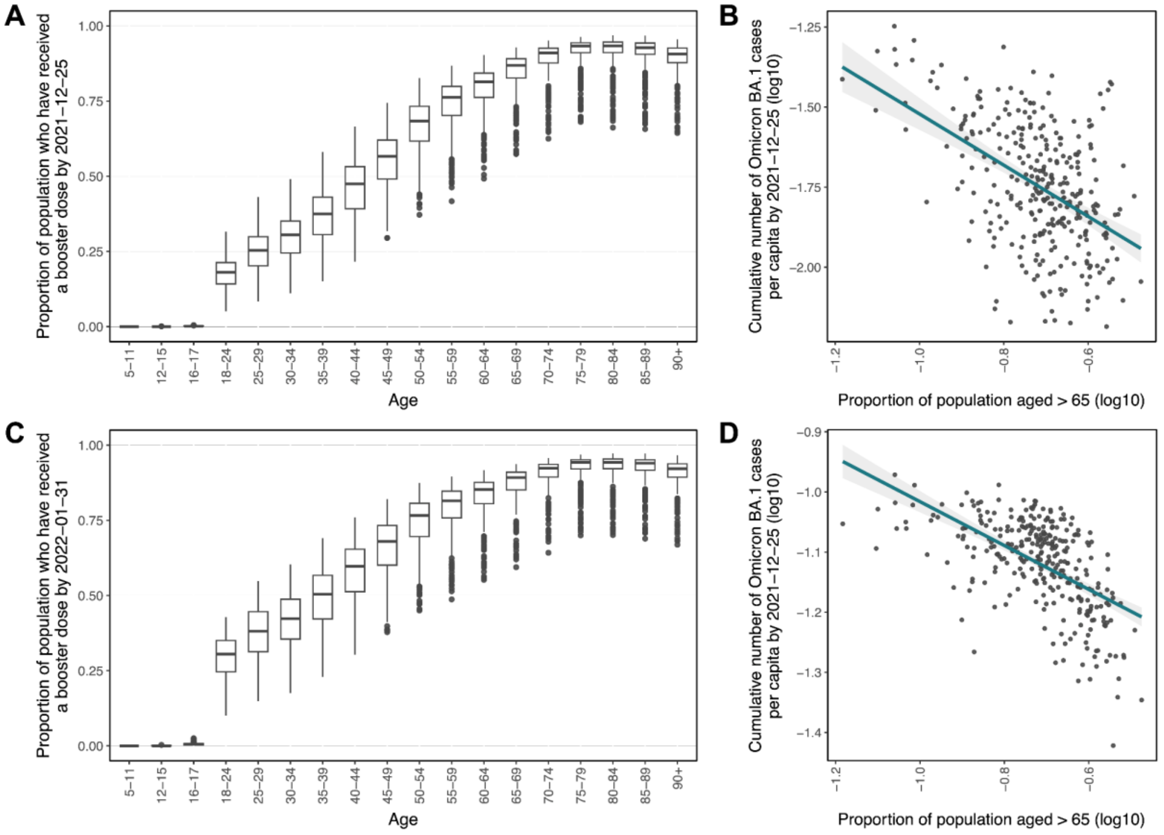

Fig. S16: Dependency of booster uptakes and cumulative Omicron BA.1 case counts on population age structure. *(A, C) Distribution of the proportion of age-specific population in each Lower Tier Local Authority (LTLA) who have received a booster dose by 25 December 2021 and 31 January 2022, respectively. Each box extends from the 25th to 75th percentile of the distribution for the corresponding age group; the midline within each box represents the median; the vertical lines represent the lower and upper limits and the dots denote the outliers. (B, D) Cumulative number of Omicron BA.1 cases per capita (log10-transformed) versus proportion of the population aged above 65 (log10-transformed). Each dot represents an LTLA. Blue lines show the least-squares fit and the shading denotes the associated 95% CI.*

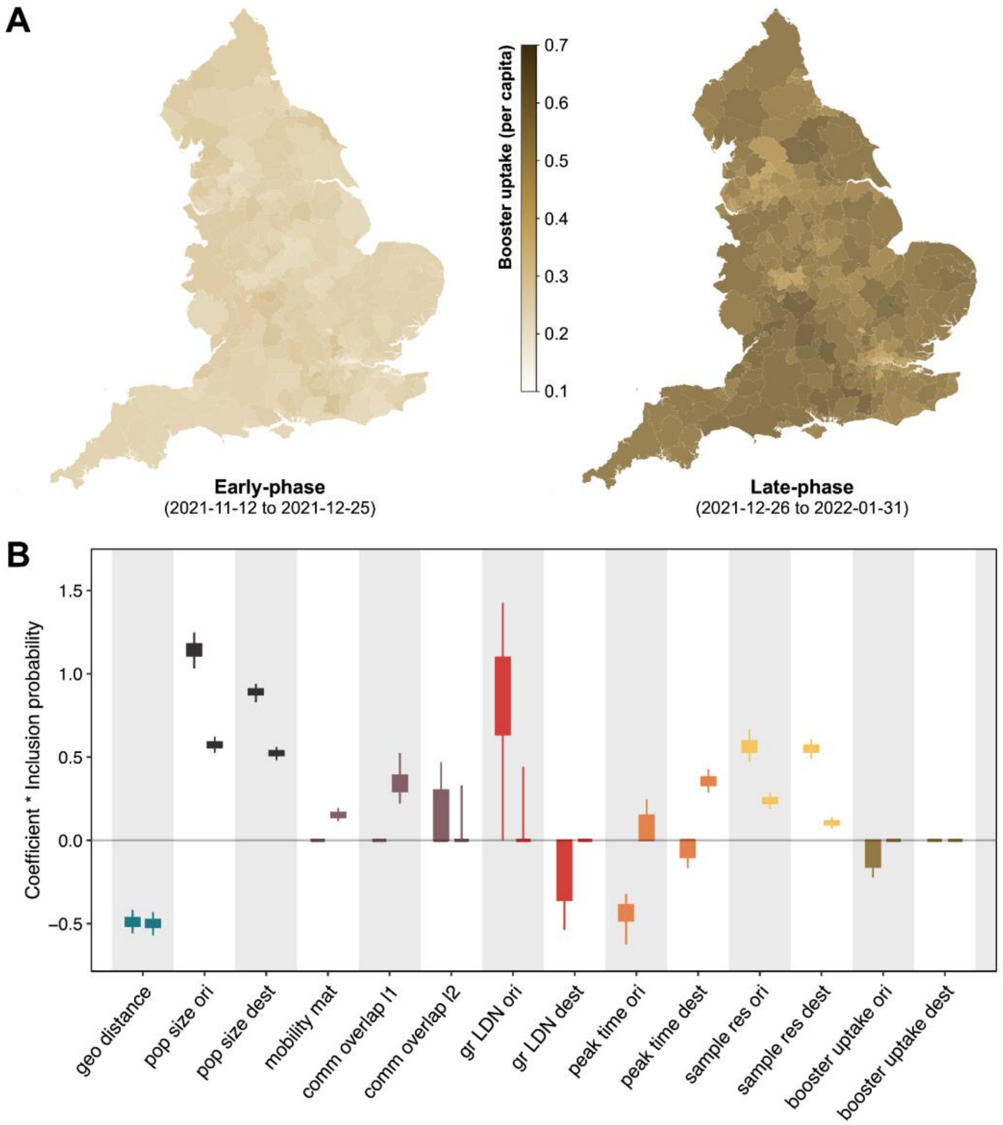

Fig. S17: Booster uptake as a predictor of Omicron BA.1 viral lineage movements in England. *(A) Map of age-corrected effective booster uptake at the Lower Tier Local Authority (LTLA) level, averaged over the early-phase (12 November 2021 to 25 December 2021) (left) and the late-phase of the epidemic (26 December 2021 to 31 January 2022) (right). The effective booster uptake is defined as the proportion of the population who would have received a booster dose having accounted for age-specific booster uptakes, assuming the national average population age structure. (B) For each predictor, the box and whiskers show the posterior distribution of the product of the log predictor coefficient and the predictor inclusion probability; the left hand value represents the expansion period estimate and the right hand value the post-expansion period estimate. Posterior distributions are coloured according to predictor type: geographic distances (geo distance, dark blue), population sizes at origin and destination (pop size ori & pop size dest, black), aggregated mobility (mobility mat, purple), mobility-based community membership level 1 and level 2 (comm overlap l1 & l2, purple), Greater London origin and destination (gr LDN ori & gr LDN dest, red), time of peak incidence at origin and destination (peak time ori & peak time dest, orange), the residual of a regression of sample size against case count regression at either origin and destination (sample res ori & sample res dest, yellow), and effective booster uptake at origin and destination (booster uptake ori & booster uptake dest, brown).*

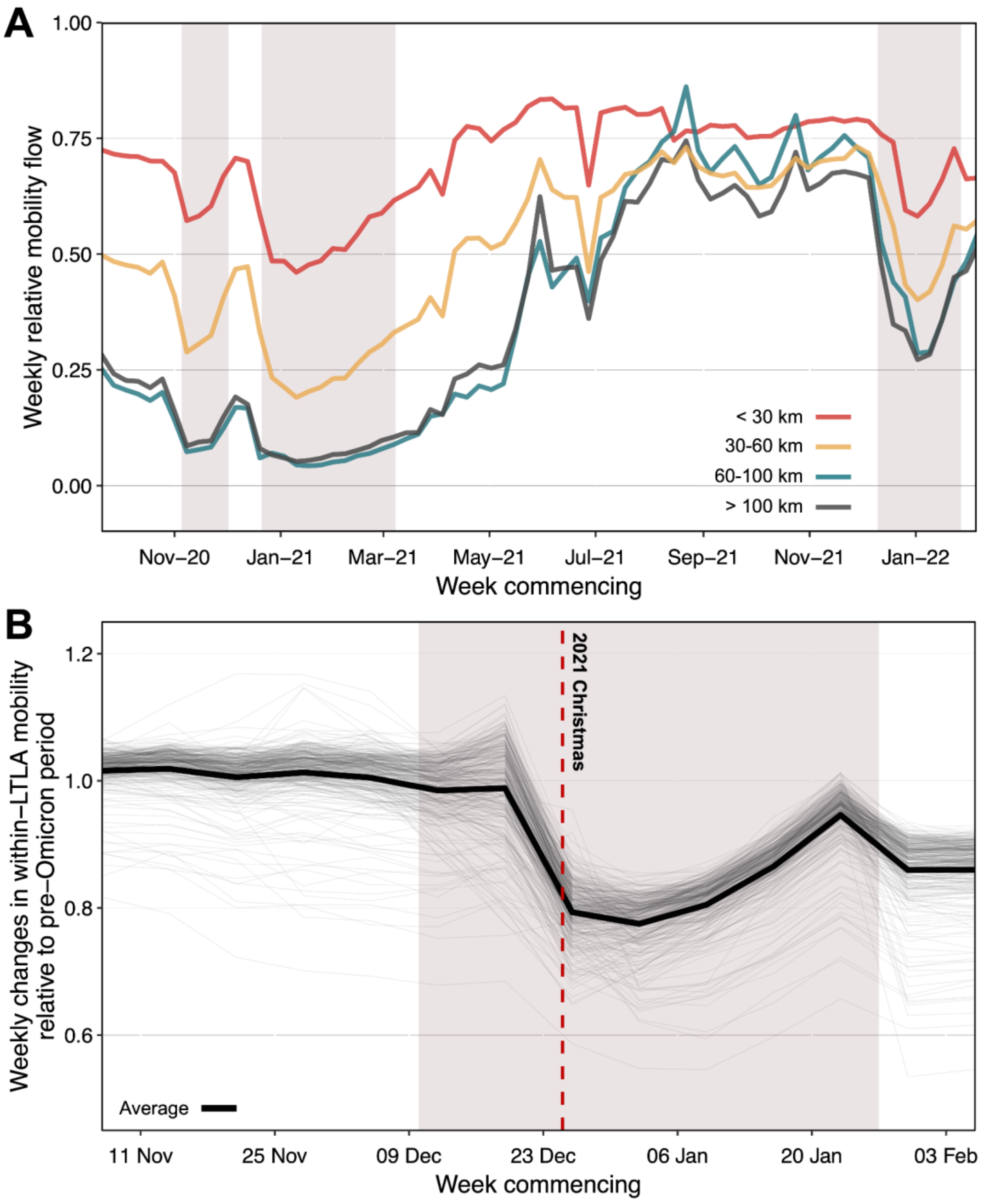

Fig. S18. Trends in human mobility across England. *(A) Weekly human mobility flows relative to pre-pandemic levels (averaged over period from 3 November 2019 to 28 December 2019) across different spatial scales (red: <30 km, yellow: 30-60 km, blue: 60-100 km, dark grey: >100 km). (B) Weekly changes in within-LTLA mobility relative to pre-Omicron levels (averaged over period from 12 September 2021 to 6 November 2021 for each LTLA individually). Thick black line represents the weekly mobility changes averaged over all LTLAs; each thin grey line represents the weekly mobility changes for a single LTLA. Vertical line shows 24th of December 2021.*

Table S1. Imputation of initial growth rate in branching process model. *Kullback-Leibler (KL) divergence values computed from comparisons of the weekly proportion of local Omicron BA.1 infections resulting from importations at different times as inferred from the phylodynamic analysis, versus predictions from branching process models with a range of initial growth rates. The initial growth rates in the branching models were imputed using estimates taken from the early growth phase of the epidemic (between 2021-12-04 and 2021-12-15), and the growth rate that minimised the KL divergence (shown in bold) was used in the best-fit model.*

Initial growth rate taken from	Kullback–Leibler divergence
**2021-12-04**	2.84
**2021-12-05**	2.04
**2021-12-06**	1.33
**2021-12-07**	1.42
**2021-12-08**	2.95
**2021-12-09**	12.54
**2021-12-10**	13.13
**2021-12-11**	8.31
**2021-12-12**	2.02
**2021-12-13**	**0.81**
**2021-12-14**	2.76
**2021-12-15**	5.17

Table S2. Imputation of initial growth rates in branching process model (sensitivity analysis using incidence estimates from the UK Office of National Statistics). *Kullback-Leibler (KL) divergence values computed from comparisons of the weekly proportion of local Omicron BA.1 infections resulting from importations at different times as inferred from the phylodynamic analysis, versus predictions from branching process models with a range of initial growth rates. The initial growth rates in the branching models were imputed using estimates taken from the early growth phase of the epidemic (between 2021-11-20 and 2021-12-20), and the growth rate that minimised the KL divergence (shown in bold) was used in the best-fit model. Note that the UK Office of National Statistics (ONS) case incidence (central) estimates are used here for the estimation of daily case growth rates, instead of case incidence data from the GOV.UK COVID-19 Dashboard.*

Initial growth rate taken from	Kullback–Leibler divergence
**2021-11-20**	5.03
**2021-11-21**	5.16
**2021-11-22**	5.29
**2021-11-23**	4.70
**2021-11-24**	4.92
**2021-11-25**	5.17
**2021-11-26**	3.77
**2021-11-27**	3.78
**2021-11-28**	3.18
**2021-11-29**	3.33
**2021-11-30**	3.9
**2021-12-01**	6.63
**2021-12-02**	17.5
**2021-12-03**	20.29
**2021-12-04**	19.00
**2021-12-05**	14.91
**2021-12-06**	12.32
**2021-12-07**	9.89
**2021-12-08**	13.77
**2021-12-09**	13.33
**2021-12-10**	13.16
**2021-12-11**	8.96
**2021-12-12**	5.60
**2021-12-13**	2.77
**2021-12-14**	1.51
**2021-12-15**	**0.7**
**2021-12-16**	1.22
**2021-12-17**	2.59
**2021-12-18**	4.18
**2021-12-19**	5.96

**2021-12-20**	7.24

Table S3*. *Predictors of viral lineage movements in England. *Summary table and descriptions of predictors considered in the discrete phylogeographic GLM analysis. For location-specific predictors (as indicated by an asterisk), we included both an origin and a destination covariate in the GLM model.*

Predictor	Domain	Time-varying	Description
Geographical distance between origin and destination	Geography	No	Geographical distance between the origin and destination calculated using the Haversine formula in km
Population size*	Demography	No	Population size at the origin/destination, from population estimates obtained by the Office of National Statistics in mid-year 2020
Aggregated mobility matrix	Human mobility	Yes	Average weekly number of trips taken between origin and destination estimated from Google COVID-19 Aggregated Mobility Research Dataset; given that the mobility flux between two locations does not in general differ from symmetry in a statistically significant manner (i.e. the magnitude of mobility flux in either direction is generally very similar for a given connection), we considered a mobility matrix that was symmetrised
Community memberships from mobility network (level-1/2)*	Human mobility	Yes	A binary variable [0,1] indicating whether the origin and destination belong to the same community at level-1/2; community structures were identified from the human mobility network as described by the aggregated mobility matrix, using the community detection algorithm Infomap (*76*, *77*), with level-1/2 corresponding to the tree-depth at which the communities were extracted; level-1 has a higher level of aggregation (fewer communities) compared to level-2 (more communities)
Greater London / non-Greater London indicator*	Geography/ Epidemiology	No	A binary variable [0,1] indicating whether the origin/destination is in the Greater London region
Timing of peak in Omicron BA.1 case incidence*	Epidemiology	No	Timing of first peak in Omicron BA.1 case incidence at the origin/destination, measured as the number of days from 1 December 2021
Sampling residuals*	Sampling	No	Residuals from a regression of sample size against Omicron BA.1 case count at origin/destination; time-invariant residuals were used due to the small number of samples in some

			locations during the post-expansion phase

Table S4. Definitions of Lower Tier Local Authorities (LTLAs). *Table of LTLAs that have been deprecated and aggregated into newly defined LTLAs, according to recent definitions used in the report of population estimates for the UK in mid-2020, compiled by the Office of National Statistics, UK.*

Most recent LTLA definition	Deprecated LTLA definition(s)
**E06000058 (Bournemouth, Christchurch and Poole)**	E06000028, E06000029, E07000048
**E06000060 (Buckinghamshire)**	E07000004, E07000005, E07000006, E07000007
**E06000059 (Dorset)**	E07000049, E07000050, E07000051, E07000052, E07000053
**E07000244 (East Suffolk)**	E07000205, E07000206
**E07000244 (West Suffolk)**	E07000201, E07000204
**E07000246 (Somerset West and Taunton)**	E07000190, E07000191

Table S6. The COVID-19 Genomics UK (COG-UK) consortium, June 2021 V4

The COVID-19 Genomics UK (COG-UK) consortium
FINAL (06-2021 V4)

Funding acquisition, Leadership and supervision, Metadata curation, Project administration, Samples and logistics, Sequencing and analysis, Software and analysis tools, and Visualisation:
Samuel C Robson [13, 84]

Funding acquisition, Leadership and supervision, Metadata curation, Project administration, Samples and logistics, Sequencing and analysis, and Software and analysis tools:
Thomas R Connor [11, 74] and Nicholas J Loman [43]

Leadership and supervision, Metadata curation, Project administration, Samples and logistics, Sequencing and analysis, Software and analysis tools, and Visualisation:
Tanya Golubchik [5]

Funding acquisition, Leadership and supervision, Metadata curation, Samples and logistics, Sequencing and analysis, and Visualisation:
Rocio T Martinez Nunez [46]

Funding acquisition, Leadership and supervision, Project administration, Samples and logistics, Sequencing and analysis, and Software and analysis tools:
David Bonsall [5]

Funding acquisition, Leadership and supervision, Project administration, Sequencing and analysis, Software and analysis tools, and Visualisation:
Andrew Rambaut [104]

Funding acquisition, Metadata curation, Project administration, Samples and logistics, Sequencing and analysis, and Software and analysis tools:
Luke B Snell [12]

Leadership and supervision, Metadata curation, Project administration, Samples and logistics, Software and analysis tools, and Visualisation:
Rich Livett [116]

Funding acquisition, Leadership and supervision, Metadata curation, Project administration, and Samples and logistics:
Catherine Ludden [20, 70]

Funding acquisition, Leadership and supervision, Metadata curation, Samples and logistics, and Sequencing and analysis:
Sally Corden [74] and Eleni Nastouli [96, 95, 30]

Funding acquisition, Leadership and supervision, Metadata curation, Sequencing and analysis, and Software and analysis tools:
Gaia Nebbia [12]

Funding acquisition, Leadership and supervision, Project administration, Samples and logistics, and Sequencing and analysis:
Ian Johnston [116]

Leadership and supervision, Metadata curation, Project administration, Samples and logistics, and Sequencing and analysis:
Katrina Lythgoe [5], M. Estee Torok [19, 20] and Ian G Goodfellow [24]

Leadership and supervision, Metadata curation, Project administration, Samples and logistics, and Visualisation:
Jacqui A Prieto [97, 82] and Kordo Saeed [97, 83]

Leadership and supervision, Metadata curation, Project administration, Sequencing and analysis, and Software and analysis tools:
David K Jackson [116]

Leadership and supervision, Metadata curation, Samples and logistics, Sequencing and analysis, and Visualisation:
Catherine Houlihan [96, 94]

Leadership and supervision, Metadata curation, Sequencing and analysis, Software and analysis tools, and Visualisation:
Dan Frampton [94, 95]

Metadata curation, Project administration, Samples and logistics, Sequencing and analysis, and Software and analysis tools:
William L Hamilton [19] and Adam A Witney [41]

Funding acquisition, Samples and logistics, Sequencing and analysis, and Visualisation:
Giselda Bucca [101]

Funding acquisition, Leadership and supervision, Metadata curation, and Project administration:
Cassie F Pope [40, 41]

Funding acquisition, Leadership and supervision, Metadata curation, and Samples and logistics:
Catherine Moore [74]

Funding acquisition, Leadership and supervision, Metadata curation, and Sequencing and analysis:
Emma C Thomson [53]

Funding acquisition, Leadership and supervision, Project administration, and Samples and logistics:
Teresa Cutino-Moguel [2], Ewan M Harrison [116, 102]

Funding acquisition, Leadership and supervision, Sequencing and analysis, and Visualisation:
Colin P Smith [101]

Leadership and supervision, Metadata curation, Project administration, and Sequencing and analysis:

Fiona Rogan [77]

Leadership and supervision, Metadata curation, Project administration, and Samples and logistics:
Shaun M Beckwith [6], Abigail Murray [6], Dawn Singleton [6], Kirstine Eastick [37], Liz A Sheridan [98], Paul Randell [99], Leigh M Jackson [105], Cristina V Ariani [116] and Sónia Gonçalves [116]

Leadership and supervision, Metadata curation, Samples and logistics, and Sequencing and analysis:
Derek J Fairley [3, 77], Matthew W Loose [18] and Joanne Watkins [74]

Leadership and supervision, Metadata curation, Samples and logistics, and Visualisation:
Samuel Moses [25, 106]

Leadership and supervision, Metadata curation, Sequencing and analysis, and Software and analysis tools:
Sam Nicholls [43], Matthew Bull [74] and Roberto Amato [116]

Leadership and supervision, Project administration, Samples and logistics, and Sequencing and analysis:
Darren L Smith [36, 65, 66]

Leadership and supervision, Sequencing and analysis, Software and analysis tools, and Visualisation:
David M Aanensen [14, 116] and Jeffrey C Barrett [116]

Metadata curation, Project administration, Samples and logistics, and Sequencing and analysis:
Beatrix Kele [2], Dinesh Aggarwal [20, 116, 70], James G Shepherd [53], Martin D Curran [71] and Surendra Parmar [71]

Metadata curation, Project administration, Sequencing and analysis, and Software and analysis tools:
Matthew D Parker [109]

Metadata curation, Samples and logistics, Sequencing and analysis, and Software and analysis tools:
Catryn Williams [74]

Metadata curation, Samples and logistics, Sequencing and analysis, and Visualisation:
Sharon Glaysher [68]

Metadata curation, Sequencing and analysis, Software and analysis tools, and Visualisation:
Anthony P Underwood [14, 116], Matthew Bashton [36, 65], Nicole Pacchiarini [74], Katie F Loveson [84] and Matthew Byott [95, 96]

Project administration, Sequencing and analysis, Software and analysis tools, and Visualisation:
Alessandro M Carabelli [20]

Funding acquisition, Leadership and supervision, and Metadata curation:
Kate E Templeton [56, 104]

Funding acquisition, Leadership and supervision, and Project administration:
Sharon J Peacock [20, 70], Thushan I de Silva [109], Dennis Wang [109], Cordelia F Langford [116] and John Sillitoe [116]

Funding acquisition, Leadership and supervision, and Samples and logistics:
Rory N Gunson [55]

Funding acquisition, Leadership and supervision, and Sequencing and analysis:
Simon Cottrell [74], Justin O'Grady [75, 103] and Dominic Kwiatkowski [116, 108]

Leadership and supervision, Metadata curation, and Project administration:
Patrick J Lillie [37]

Leadership and supervision, Metadata curation, and Samples and logistics:
Nicholas Cortes [33], Nathan Moore [33], Claire Thomas [33], Phillipa J Burns [37], Tabitha W Mahungu [80] and Steven Liggett [86]

Leadership and supervision, Metadata curation, and Sequencing and analysis:
Angela H Beckett [13, 81] and Matthew TG Holden [73]

Leadership and supervision, Project administration, and Samples and logistics:
Lisa J Levett [34], Husam Osman [70, 35] and Mohammed O Hassan-Ibrahim [99]

Leadership and supervision, Project administration, and Sequencing and analysis:
David A Simpson [77]

Leadership and supervision, Samples and logistics, and Sequencing and analysis:
Meera Chand [72], Ravi K Gupta [102], Alistair C Darby [107] and Steve Paterson [107]

Leadership and supervision, Sequencing and analysis, and Software and analysis tools:
Oliver G Pybus [23], Erik M Volz [39], Daniela de Angelis [52], David L Robertson [53], Andrew J Page [75] and Inigo Martincorena [116]

Leadership and supervision, Sequencing and analysis, and Visualisation:
Louise Aigrain [116] and Andrew R Bassett [116]

Metadata curation, Project administration, and Samples and logistics:
Nick Wong [50], Yusri Taha [89], Michelle J Erkiert [99] and Michael H Spencer Chapman [116, 102]

Metadata curation, Project administration, and Sequencing and analysis:
Rebecca Dewar [56] and Martin P McHugh [56, 111]

Metadata curation, Project administration, and Software and analysis tools:
Siddharth Mookerjee [38, 57]

Metadata curation, Project administration, and Visualisation:
Stephen Aplin [97], Matthew Harvey [97], Thea Sass [97], Helen Umpleby [97] and Helen Wheeler [97]

Metadata curation, Samples and logistics, and Sequencing and analysis:
James P McKenna [3], Ben Warne [9], Joshua F Taylor [22], Yasmin Chaudhry [24], Rhys Izuagbe [24], Aminu S Jahun [24], Gregory R Young [36, 65], Claire McMurray [43], Clare M McCann [65, 66], Andrew Nelson [65, 66] and Scott Elliott [68]

Metadata curation, Samples and logistics, and Visualisation:
Hannah Lowe [25]

Metadata curation, Sequencing and analysis, and Software and analysis tools:
Anna Price [11], Matthew R Crown [65], Sara Rey [74], Sunando Roy [96] and Ben Temperton [105]

Metadata curation, Sequencing and analysis, and Visualisation:
Sharif Shaaban [73] and Andrew R Hesketh [101]

Project administration, Samples and logistics, and Sequencing and analysis:
Kenneth G Laing [41], Irene M Monahan [41] and Judith Heaney [95, 96, 34]

Project administration, Samples and logistics, and Visualisation:
Emanuela Pelosi [97], Siona Silviera [97] and Eleri Wilson-Davies [97]

Samples and logistics, Software and analysis tools, and Visualisation:
Helen Fryer [5]

Sequencing and analysis, Software and analysis tools, and Visualization:
Helen Adams [4], Louis du Plessis [23], Rob Johnson [39], William T Harvey [53, 42], Joseph Hughes [53], Richard J Orton [53], Lewis G Spurgin [59], Yann Bourgeois [81], Chris Ruis [102], Áine O'Toole [104], Marina Gourtovaia [116] and Theo Sanderson [116]

Funding acquisition, and Leadership and supervision:
Christophe Fraser [5], Jonathan Edgeworth [12], Judith Breuer [96, 29], Stephen L Michell [105] and John A Todd [115]

Funding acquisition, and Project administration:
Michaela John [10] and David Buck [115]

Leadership and supervision, and Metadata curation:
Kavitha Gajee [37] and Gemma L Kay [75]

Leadership and supervision, and Project administration:
David Heyburn [74]

Leadership and supervision, and Samples and logistics:
Themoula Charalampous [12, 46], Adela Alcolea-Medina [32, 112], Katie Kitchman [37], Alan McNally [43, 93], David T Pritchard [50], Samir Dervisevic [58], Peter Muir [70], Esther Robinson [70, 35], Barry B Vipond [70], Newara A Ramadan [78], Christopher Jeanes [90], Danni Weldon [116], Jana Catalan [118] and Neil Jones [118]

Leadership and supervision, and Sequencing and analysis:
Ana da Silva Filipe [53], Chris Williams [74], Marc Fuchs [77], Julia Miskelly [77], Aaron R Jeffries [105], Karen Oliver [116] and Naomi R Park [116]

Metadata curation, and Samples and logistics:
Amy Ash [1], Cherian Koshy [1], Magdalena Barrow [7], Sarah L Buchan [7], Anna Mantzouratou [7], Gemma Clark [15], Christopher W Holmes [16], Sharon Campbell [17], Thomas Davis [21], Ngee Keong Tan [22], Julianne R Brown [29], Kathryn A Harris [29, 2], Stephen P Kidd [33], Paul R Grant [34], Li Xu-McCrae [35], Alison Cox [38, 63], Pinglawathee Madona [38, 63], Marcus Pond [38, 63], Paul A Randell [38, 63], Karen T Withell [48], Cheryl Williams [51], Clive Graham [60], Rebecca Denton-Smith [62], Emma Swindells [62], Robyn Turnbull [62], Tim J Sloan [67], Andrew Bosworth [70, 35], Stephanie Hutchings [70], Hannah M Pymont [70], Anna Casey [76], Liz Ratcliffe [76], Christopher R Jones [79, 105], Bridget A Knight [79, 105], Tanzina Haque [80], Jennifer Hart [80], Dianne Irish-Tavares [80], Eric Witele [80], Craig Mower [86], Louisa K Watson [86], Jennifer Collins [89], Gary Eltringham [89], Dorian Crudgington [98], Ben Macklin [98], Miren Iturriza-Gomara [107], Anita O Lucaci [107] and Patrick C McClure [113]

Metadata curation, and Sequencing and analysis:
Matthew Carlile [18], Nadine Holmes [18], Christopher Moore [18], Nathaniel Storey [29], Stefan Rooke [73], Gonzalo Yebra [73], Noel Craine [74], Malorie Perry [74], Nabil-Fareed Alikhan [75], Stephen Bridgett [77], Kate F Cook [84], Christopher Fearn [84], Salman Goudarzi [84], Ronan A Lyons [88], Thomas Williams [104], Sam T Haldenby [107], Jillian Durham [116] and Steven Leonard [116]

Metadata curation, and Software and analysis tools:
Robert M Davies [116]

Project administration, and Samples and logistics:
Rahul Batra [12], Beth Blane [20], Moira J Spyer [30, 95, 96], Perminder Smith [32, 112], Mehmet Yavus [85, 109], Rachel J Williams [96], Adhyana IK Mahanama [97], Buddhini Samaraweera [97], Sophia T Girgis [102], Samantha E Hansford [109], Angie Green [115], Charlotte Beaver [116], Katherine L Bellis [116, 102], Matthew J Dorman [116], Sally Kay [116], Liam Prestwood [116] and Shavanthi Rajatileka [116]

Project administration, and Sequencing and analysis:
Joshua Quick [43]

Project administration, and Software and analysis tools:
Radoslaw Poplawski [43]

Samples and logistics, and Sequencing and analysis:
Nicola Reynolds [8], Andrew Mack [11], Arthur Morriss [11], Thomas Whalley [11], Bindi Patel [12], Iliana Georgana [24], Myra Hosmillo [24], Malte L Pinckert [24], Joanne Stockton [43], John H Henderson [65], Amy Hollis [65], William Stanley [65], Wen C Yew [65], Richard Myers [72], Alicia Thornton [72], Alexander Adams [74], Tara Annett [74], Hibo Asad [74], Alec Birchley [74], Jason Coombes [74], Johnathan M Evans [74], Laia Fina [74], Bree Gatica-Wilcox [74], Lauren Gilbert [74], Lee Graham [74], Jessica Hey [74], Ember Hilvers [74], Sophie Jones [74], Hannah Jones [74], Sara Kumziene-Summerhayes [74], Caoimhe McKerr [74], Jessica Powell [74], Georgia Pugh [74], Sarah Taylor [74], Alexander J Trotter [75], Charlotte A Williams [96], Leanne M Kermack [102], Benjamin H Foulkes [109], Marta Gallis [109], Hailey R Hornsby [109], Stavroula F Louka [109], Manoj Pohare [109], Paige Wolverson [109], Peijun Zhang [109], George MacIntyre-Cockett [115], Amy Trebes [115], Robin J Moll [116], Lynne Ferguson [117], Emily J Goldstein [117], Alasdair Maclean [117] and Rachael Tomb [117]

Samples and logistics, and Software and analysis tools:
Igor Starinskij [53]

Sequencing and analysis, and Software and analysis tools:
Laura Thomson [5], Joel Southgate [11, 74], Moritz UG Kraemer [23], Jayna Raghwani [23], Alex E Zarebski [23], Olivia Boyd [39], Lily Geidelberg [39], Chris J Illingworth [52], Chris Jackson [52], David Pascall [52], Sreenu Vattipally [53], Timothy M Freeman [109], Sharon N Hsu [109], Benjamin B Lindsey [109], Keith James [116], Kevin Lewis [116], Gerry Tonkin-Hill [116] and Jaime M Tovar-Corona [116]

Sequencing and analysis, and Visualisation:
MacGregor Cox [20]

Software and analysis tools, and Visualisation:
Khalil Abudahab [14, 116], Mirko Menegazzo [14], Ben EW Taylor MEng [14, 116], Corin A Yeats [14], Afrida Mukaddas [53], Derek W Wright [53], Leonardo de Oliveira Martins [75], Rachel Colquhoun [104], Verity Hill [104], Ben Jackson [104], JT McCrone [104], Nathan Medd [104], Emily Scher [104] and Jon-Paul Keatley [116]

Leadership and supervision:
Tanya Curran [3], Sian Morgan [10], Patrick Maxwell [20], Ken Smith [20], Sahar Eldirdiri [21], Anita Kenyon [21], Alison H Holmes [38, 57], James R Price [38, 57], Tim Wyatt [69], Alison E Mather [75], Timofey Skvortsov [77] and John A Hartley [96]

Metadata curation:
Martyn Guest [11], Christine Kitchen [11], Ian Merrick [11], Robert Munn [11], Beatrice Bertolusso [33], Jessica Lynch [33], Gabrielle Vernet [33], Stuart Kirk [34], Elizabeth Wastnedge [56], Rachael Stanley [58], Giles Idle [64], Declan T Bradley [69, 77], Nicholas F Killough [69], Jennifer Poyner [79] and Matilde Mori [110]

Project administration:

Owen Jones [11], Victoria Wright [18], Ellena Brooks [20], Carol M Churcher [20], Laia Delgado Callico [20], Mireille Fragakis [20], Katerina Galai [20, 70], Andrew Jermy [20], Sarah Judges [20], Anna Markov [20], Georgina M McManus [20], Kim S Smith [20], Peter M D Thomas-McEwen [20], Elaine Westwick [20], Stephen W Attwood [23], Frances Bolt [38, 57], Alisha Davies [74], Elen De Lacy [74], Fatima Downing [74], Sue Edwards [74], Lizzie Meadows [75], Sarah Jeremiah [97], Nikki Smith [109] and Luke Foulser [116]

Samples and logistics:

Amita Patel [12], Louise Berry [15], Tim Boswell [15], Vicki M Fleming [15], Hannah C Howson-Wells [15], Amelia Joseph [15], Manjinder Khakh [15], Michelle M Lister [15], Paul W Bird [16], Karlie Fallon [16], Thomas Helmer [16], Claire L McMurray [16], Mina Odedra [16], Jessica Shaw [16], Julian W Tang [16], Nicholas J Willford [16], Victoria Blakey [17], Veena Raviprakash [17], Nicola Sheriff [17], Lesley-Anne Williams [17], Theresa Feltwell [20], Luke Bedford [26], James S Cargill [27], Warwick Hughes [27], Jonathan Moore [28], Susanne Stonehouse [28], Laura Atkinson [29], Jack CD Lee [29], Dr Divya Shah [29], Natasha Ohemeng-Kumi [32, 112], John Ramble [32, 112], Jasveen Sehmi [32, 112], Rebecca Williams [33], Wendy Chatterton [34], Monika Pusok [34], William Everson [37], Anibolina Castigador [44], Emily Macnaughton [44], Kate El Bouzidi [45], Temi Lampejo [45], Malur Sudhanva [45], Cassie Breen [47], Graciela Sluga [48], Shazaad SY Ahmad [49, 70], Ryan P George [49], Nicholas W Machin [49, 70], Debbie Binns [50], Victoria James [50], Rachel Blacow [55], Lindsay Coupland [58], Louise Smith [59], Edward Barton [60], Debra Padgett [60], Garren Scott [60], Aidan Cross [61], Mariyam Mirfenderesky [61], Jane Greenaway [62], Kevin Cole [64], Phillip Clarke [67], Nichola Duckworth [67], Sarah Walsh [67], Kelly Bicknell [68], Robert Impey [68], Sarah Wyllie [68], Richard Hopes [70], Chloe Bishop [72], Vicki Chalker [72], Ian Harrison [72], Laura Gifford [74], Zoltan Molnar [77], Cressida Auckland [79], Cariad Evans [85, 109], Kate Johnson [85, 109], David G Partridge [85, 109], Mohammad Raza [85, 109], Paul Baker [86], Stephen Bonner [86], Sarah Essex [86], Leanne J Murray [86], Andrew I Lawton [87], Shirelle Burton-Fanning [89], Brendan AI Payne [89], Sheila Waugh [89], Andrea N Gomes [91], Maimuna Kimuli [91], Darren R Murray [91], Paula Ashfield [92], Donald Dobie [92], Fiona Ashford [93], Angus Best [93], Liam Crawford [93], Nicola Cumley [93], Megan Mayhew [93], Oliver Megram [93], Jeremy Mirza [93], Emma Moles-Garcia [93], Benita Percival [93], Megan Driscoll [96], Leah Ensell [96], Helen L Lowe [96], Laurentiu Maftei [96], Matteo Mondani [96], Nicola J Chaloner [99], Benjamin J Cogger [99], Lisa J Easton [99], Hannah Huckson [99], Jonathan Lewis [99], Sarah Lowdon [99], Cassandra S Malone [99], Florence Munemo [99], Manasa Mutingwende [99], Roberto Nicodemi [99], Olga Podplomyk [99], Thomas Somassa [99], Andrew Beggs [100], Alex Richter [100], Claire Cormie [102], Joana Dias [102], Sally Forrest [102], Ellen E Higginson [102], Mailis Maes [102], Jamie Young [102], Rose K Davidson [103], Kathryn A Jackson [107], Alexander J Keeley [109], Jonathan Ball [113], Timothy Byaruhanga [113], Joseph G Chappell [113], Jayasree Dey [113], Jack D Hill [113], Emily J Park [113], Arezou Fanaie [114], Rachel A Hilson [114], Geraldine Yaze [114] and Stephanie Lo [116]

Sequencing and analysis:

Safiah Afifi [10], Robert Beer [10], Joshua Maksimovic [10], Kathryn McCluggage [10], Karla Spellman [10], Catherine Bresner [11], William Fuller [11], Angela Marchbank [11], Trudy Workman [11], Ekaterina Shelest [13, 81], Johnny Debebe [18], Fei Sang [18], Sarah Francois [23], Bernardo Gutierrez [23], Tetyana I Vasylyeva [23], Flavia Flaviani [31], Manon Ragonnet-Cronin [39], Katherine L Smollett [42], Alice Broos [53], Daniel Mair [53], Jenna Nichols [53], Kyriaki Nomikou [53], Lily Tong [53], Ioulia Tsatsani [53], Sarah O'Brien [54], Steven Rushton [54], Roy Sanderson [54], Jon Perkins [55], Seb Cotton [56], Abbie Gallagher [56], Elias Allara [70, 102], Clare Pearson [70, 102], David Bibby [72], Gavin Dabrera [72], Nicholas Ellaby [72], Eileen Gallagher [72], Jonathan Hubb [72], Angie Lackenby [72], David Lee [72], Nikos Manesis [72], Tamyo Mbisa [72], Steven Platt [72], Katherine A Twohig [72], Mari Morgan [74], Alp Aydin [75], David J Baker [75], Ebenezer Foster-Nyarko [75], Sophie J Prosolek [75], Steven Rudder [75], Chris Baxter [77], Sílvia F Carvalho [77], Deborah Lavin [77], Arun Mariappan [77], Clara Radulescu [77], Aditi Singh [77], Miao Tang [77], Helen Morcrette [79], Nadua Bayzid [96], Marius Cotic [96], Carlos E Balcazar [104], Michael D Gallagher [104], Daniel Maloney [104], Thomas D Stanton [104], Kathleen A Williamson [104], Robin Manley [105], Michelle L Michelsen [105], Christine M Sambles [105], David J Studholme [105], Joanna Warwick-Dugdale [105], Richard Eccles [107], Matthew Gemmell [107], Richard Gregory [107], Margaret Hughes [107], Charlotte Nelson [107], Lucille Rainbow [107], Edith E Vamos [107], Hermione J Webster [107], Mark Whitehead [107], Claudia Wierzbicki [107], Adrienn Angyal [109], Luke R Green [109], Max Whiteley [109], Emma Betteridge [116], Iraad F Bronner [116], Ben W Farr [116], Scott Goodwin [116], Stefanie V Lensing [116], Shane

A McCarthy [116, 102], Michael A Quail [116], Diana Rajan [116], Nicholas M Redshaw [116], Carol Scott [116], Lesley Shirley [116] and Scott AJ Thurston [116]

Software and analysis tools:
Will Rowe [43], Amy Gaskin [74], Thanh Le-Viet [75], James Bonfield [116], Jennifier Liddle [116] and Andrew Whitwham [116]

1 Barking, Havering and Redbridge University Hospitals NHS Trust, **2** Barts Health NHS Trust, **3** Belfast Health & Social Care Trust, **4** Betsi Cadwaladr University Health Board, **5** Big Data Institute, Nuffield Department of Medicine, University of Oxford, **6** Blackpool Teaching Hospitals NHS Foundation Trust, **7** Bournemouth University, **8** Cambridge Stem Cell Institute, University of Cambridge, **9** Cambridge University Hospitals NHS Foundation Trust, **10** Cardiff and Vale University Health Board, **11** Cardiff University, **12** Centre for Clinical Infection and Diagnostics Research, Department of Infectious Diseases, Guy's and St Thomas' NHS Foundation Trust, **13** Centre for Enzyme Innovation, University of Portsmouth, **14** Centre for Genomic Pathogen Surveillance, University of Oxford, **15** Clinical Microbiology Department, Queens Medical Centre, Nottingham University Hospitals NHS Trust, **16** Clinical Microbiology, University Hospitals of Leicester NHS Trust, **17** County Durham and Darlington NHS Foundation Trust, **18** Deep Seq, School of Life Sciences, Queens Medical Centre, University of Nottingham, **19** Department of Infectious Diseases and Microbiology, Cambridge University Hospitals NHS Foundation Trust, **20** Department of Medicine, University of Cambridge, **21** Department of Microbiology, Kettering General Hospital, **22** Department of Microbiology, South West London Pathology, **23** Department of Zoology, University of Oxford, **24** Division of Virology, Department of Pathology, University of Cambridge, **25** East Kent Hospitals University NHS Foundation Trust, **26** East Suffolk and North Essex NHS Foundation Trust, **27** East Sussex Healthcare NHS Trust**, 28** Gateshead Health NHS Foundation Trust, **29** Great Ormond Street Hospital for Children NHS Foundation Trust, **30** Great Ormond Street Institute of Child Health (GOS ICH), University College London (UCL), **31** Guy's and St. Thomas' Biomedical Research Centre, **32** Guy's and St. Thomas' NHS Foundation Trust, **33** Hampshire Hospitals NHS Foundation Trust, **34** Health Services Laboratories, **35** Heartlands Hospital, Birmingham, **36** Hub for Biotechnology in the Built Environment, Northumbria University, **37** Hull University Teaching Hospitals NHS Trust, **38** Imperial College Healthcare NHS Trust, **39** Imperial College London, **40** Infection Care Group, St George's University Hospitals NHS Foundation Trust, **41** Institute for Infection and Immunity, St George's University of London, **42** Institute of Biodiversity, Animal Health & Comparative Medicine, **43** Institute of Microbiology and Infection, University of Birmingham, **44** Isle of Wight NHS Trust, **45** King's College Hospital NHS Foundation Trust, **46** King's College London, **47** Liverpool Clinical Laboratories, **48** Maidstone and Tunbridge Wells NHS Trust, **49** Manchester University NHS Foundation Trust, **50** Microbiology Department, Buckinghamshire Healthcare NHS Trust, **51** Microbiology, Royal Oldham Hospital, **52** MRC Biostatistics Unit, University of Cambridge, **53** MRC-University of Glasgow Centre for Virus Research, **54** Newcastle University, **55** NHS Greater Glasgow and Clyde, **56** NHS Lothian, **57** NIHR Health Protection Research Unit in HCAI and AMR, Imperial College London, **58** Norfolk and Norwich University Hospitals NHS Foundation Trust, **59** Norfolk County Council, **60** North Cumbria Integrated Care NHS Foundation Trust, **61** North Middlesex University Hospital NHS Trust, **62** North Tees and Hartlepool NHS Foundation Trust, **63** North West London Pathology, **64** Northumbria Healthcare NHS Foundation Trust, **65** Northumbria University, **66** NU-OMICS, Northumbria University, **67** Path Links, Northern Lincolnshire and Goole NHS Foundation Trust, **68** Portsmouth Hospitals University NHS Trust, **69** Public Health Agency, Northern Ireland, **70** Public Health England, **71** Public Health England, Cambridge, **72** Public Health England, Colindale, **73** Public Health Scotland, **74** Public Health Wales, **75** Quadram Institute Bioscience, **76** Queen Elizabeth Hospital, Birmingham, **77** Queen's University Belfast, **78** Royal Brompton and Harefield Hospitals, **79** Royal Devon and Exeter NHS Foundation Trust, **80** Royal Free London NHS Foundation Trust, **81** School of Biological Sciences, University of Portsmouth, **82** School of Health Sciences, University of Southampton, **83** School of Medicine, University of Southampton, **84** School of Pharmacy & Biomedical Sciences, University of Portsmouth, **85** Sheffield Teaching Hospitals NHS Foundation Trust, **86** South Tees Hospitals NHS Foundation Trust, **87** Southwest Pathology Services, **88** Swansea University, **89** The Newcastle upon Tyne Hospitals NHS Foundation Trust, **90** The Queen Elizabeth Hospital King's Lynn NHS Foundation Trust, **91** The Royal Marsden NHS Foundation Trust, **92** The

Royal Wolverhampton NHS Trust, **93** Turnkey Laboratory, University of Birmingham, **94** University College London Division of Infection and Immunity**, 95** University College London Hospital Advanced Pathogen Diagnostics Unit**, 96** University College London Hospitals NHS Foundation Trust, **97** University Hospital Southampton NHS Foundation Trust, **98** University Hospitals Dorset NHS Foundation Trust, **99** University Hospitals Sussex NHS Foundation Trust, **100** University of Birmingham, **101** University of Brighton, **102** University of Cambridge, **103** University of East Anglia, **104** University of Edinburgh, **105** University of Exeter, **106** University of Kent, **107** University of Liverpool, **108** University of Oxford, **109** University of Sheffield, **110** University of Southampton, **111** University of St Andrews, **112** Viapath, Guy's and St Thomas' NHS Foundation Trust, and King's College Hospital NHS Foundation Trust, **113** Virology, School of Life Sciences, Queens Medical Centre, University of Nottingham, **114** Watford General Hospital, **115** Wellcome Centre for Human Genetics, Nuffield Department of Medicine, University of Oxford, **116** Wellcome Sanger Institute, **117** West of Scotland Specialist Virology Centre, NHS Greater Glasgow and Clyde, **118** Whittington Health NHS Trust
